# *Antrodia salmonea* induces apoptosis and enhances cytoprotective autophagy in colon cancer cells

**DOI:** 10.18632/aging.203019

**Published:** 2021-05-24

**Authors:** Hsin-Ling Yang, Hui-Wen Liu, Sirjana Shrestha, Varadharajan Thiyagarajan, Hui-Chi Huang, You-Cheng Hseu

**Affiliations:** 1 Institute of Nutrition, College of Health Care, China Medical University, Taichung 40402, Taiwan; 2 Department of Chinese Pharmaceutical Sciences and Chinese Medicine Resources, College of Chinese Medicine, China Medical University, Taichung 40402, Taiwan; 3 Department of Cosmeceutics, College of Pharmacy, China Medical University, Taichung 40402, Taiwan; 4 Department of Health and Nutrition Biotechnology, Asia University, Taichung 41354, Taiwan; 5 Chinese Medicine Research Center, China Medical University, Taichung 40402, Taiwan

**Keywords:** *Antrodia salmonea*, colon cancer, apoptosis, autophagy, ROS

## Abstract

A traditional Chinese medicinal fungus, *Antrodia salmonea* (AS), with antioxidant properties is familiar in Taiwan but anti-cancer activity of AS in human colon cancer is ambiguous. Hence, we explored the anti-cancer activity of AS in colon cancer cells. 3-(4,5-dimethylthiazol-2-yl)-2,5-diphenyltetrazolium bromide (MTT) assay revealed that AS showed a remarkable effect on cell viability in colon cancer cells; SW620, HCT116, and HT29. Annexin V/propidium iodide (PI) stained cells indicated that AS induced both early/late apoptosis in SW620 cells. Additionally, cells treated with AS induced caspase-3 activation, poly (ADP-ribose) polymerase (PARP) cleavage, mitochondrial dysfunction, and Bcl-2 associated X (Bax)/B-cell lymphoma (Bcl-2) dysregulation. Microtubule- associated protein 1A/1B-light chain 3B (LC3-II) accumulation, sequestosome 1 (p62/SQSTM1) activation, autophagy related 4B cysteine peptidase (ATG4B) inactivation, acidic vesicular organelles (AVOs) formation, and Beclin-1/Bcl-2 dysregulation revealed that AS-induced autophagy. Interestingly, cells pretreated with 3-methyladenine (3-MA) strengthened AS-induced caspase-3/apoptosis. Suppression of apoptosis by z-Val-Ala-Asp fluoromethyl ketone (Z-VAD-FMK) did not however block AS-induced autophagy, suggesting that autophagy was not attenuated by the AS-induced apoptosis. Application of *N*-acetylcysteine (NAC) prevented AS-induced cell death, caspase-3 activation, LC3-II accumulation, and AVOs formation, indicating that AS-induced apoptosis and autophagy was mediated by reactive oxygen species (ROS). Furthermore, AS-induced cytoprotective autophagy and apoptosis through extracellular signal-regulated kinase (ERK) signaling cascades. Moreover, *in vivo* data disclosed that AS inhibited colitis-associated tumorigenesis in azoxymethane (AOM)-dextran sodium sulphate (DSS)-treated mice. For the first time, we report the anti-cancer properties of this potentially advantageous mushroom for the treatment of human colon cancer.

## INTRODUCTION

Colorectal cancer (CRC) is the third most deadly and fourth most frequently identified cancer in the world [1]. Cancers of the colon emerge from the epithelial cells of colon which line the lumen of the organ [2]. One of the major risk factors for the occurrence of colorectal cancer is the consumption of high fat diet [3]. Likewise, environmental and genetic factors also play crucial roles in the pathogenesis of disease [4]. Even though chemotherapy based on 5-FU remains the first-line chemotherapy treatment for advanced CRC, irrespective of the advent of new chemotherapy drugs, chemoresistance and extreme side effects however have been the biggest barrier to enhance the effectiveness of combined therapy [5, 6]. Furthermore, Cetuximab and Panitumumab are two distinct monoclonal antibodies that target and bind with the epidermal growth factor receptor (EGFR), thus reducing its activation and dimerization. Both boost the consequences of metastatic colorectal cancer (mCRC) patients either in monotherapy or in conjunction with chemotherapy [7]. However, 80–95% of patients with mCRC reported skin toxicities by the application of these monotherapies [8]. Hence, there is a clear need to develop innovative anti-cancer agents with lesser side effects in treatment of the colon cancer.

Apoptosis is a process that is highly coordinated and closely regulated and is considered as one of the most frequent forms of programmed death of cells. Apoptosis requires a series of biochemical modifications directing to the cell death [9]. There are many biochemical modifications in apoptotic cells, for instance, protein cleavage, protein cross-linking, and DNA disintegration which result in distinctive structural pathology [10]. Several cellular events are triggered by apoptotic signals, which include extensive reactive oxygen species (ROS) generation, mitochondrial damage, and death receptor expression [11]. As an insufficient amount of apoptosis contributes to cancer, the focus in the treatment of cancer is on targeting apoptosis. Caspases (−8, −9, and −3) are initiated due to extrinsic (death receptor) as well as intrinsic (mitochondrial) apoptotic pathways, which cleave different substrates that result in DNA fragmentation and eventually lead to cell death [12]. The major issues with cancer cells are cells grow and proliferate out of control and become resistance to apoptosis [13]. Numerous evidence suggest that using chemical compounds or pro-apoptotic agents to induce apoptosis can effectively minimize the spread of cancer [14–16]. Therefore, apoptosis induction would be a simple approach in treatment of any types of cancer. Researches have concentrated on exploring chemical or biological substances that may cause apoptosis and are non-toxic to ordinary cells at the same time.

Autophagy, also known as cellular self-digestion is a vital phenomenon accompanied with the turnover of long-lasting proteins and organelles along with recycling of substances to continue cellular components quality [17]. In case if there is no apoptosis, then autophagy induces a type of cell death which is referred as autophagic cell death also called as Type II programmed cell death that differs from Type I programmed cell death i.e. apoptosis [18]. In the regulation of autophagy, many molecular and cellular signaling pathways are involved, such as microtubule-associated light chain 3 (LC3), autophagy related 5 (ATG5)/autophagy related 7 (ATG7), rapamycin mammalian target (mTOR), and Beclin-1 [19]. Depending on the cellular background, the functional connection amongst autophagy and apoptosis is complicated. Too much production of ROS seriously destructs DNA and proteins and diminishes the mitochondrial membrane potential (ΔΨm) resulting in autophagy and apoptosis [20]. Therefore, the main goal of tumor chemoprevention is to stop the promotion and activation of cancer cells and to induce apoptosis/autophagy.

Since long time *Antrodia salmonea* (AS) or *Taiwanofungus salmoneus* had been in practice as a conventional Chinese medicine in treating different complications like drug overdose, food poisoning, hypertension, stomach pain, skin irritation, diarrhea, and some cancers [21]. Several natural compounds have so far been isolated from AS and its basic chemical constituents and unprocessed extracts showed antioxidant effects [22]. AS suppressed TNF-α-induced angiogenesis/atherogenesis by regulating NFκB and HO-1/Nrf2 signaling pathways [23]. AS had shown its anti-cancer effects in ovarian cancer [24], triple negative breast cancer [25], and promyelocytic leukemia [26]. AS had shown strong antioxidant properties and thus contributes for protecting human from atherogenesis [27]. As far as we know, the pharmacological or biological activities of AS against colon cancer has not yet been carried out. Therefore, this study is focused to find out the effectiveness of AS against human colon cancer cells. Here in this research, we exhibited the potential of a fermented broth of AS produced from submerged culture in case of human colon cancer cells. In SW620 cells, AS decreased cell viability and induced apoptosis and cytoprotective autophagy via intracellular ROS and interruption of the signaling pathways for ERK and AKT.

## RESULTS

### *Antrodia salmonea* treatment inhibits cell viability of colon cancer cells and induces ROS generation in SW620 cells

**Figure 1:**
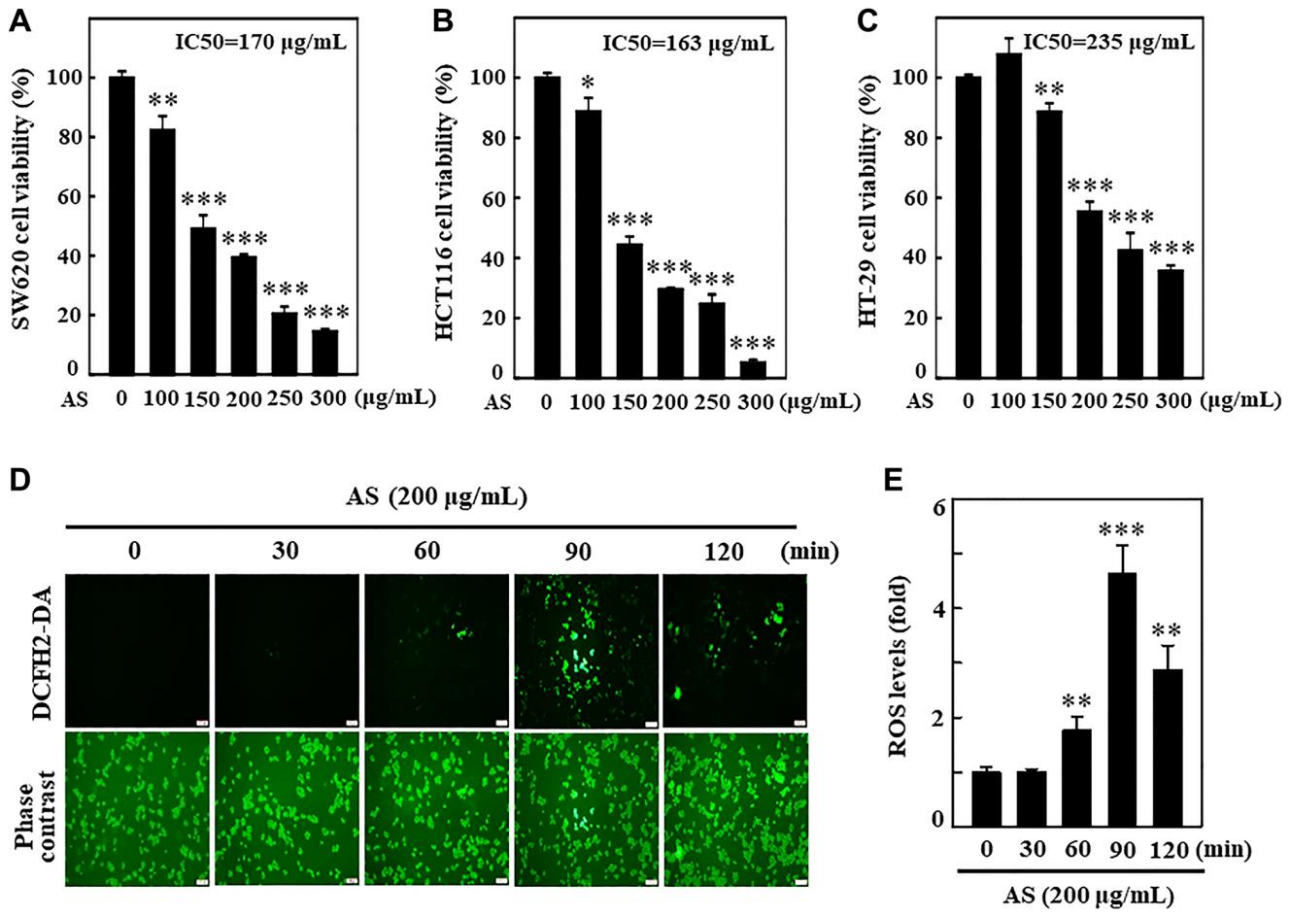
***Antrodia salmonea* (AS) inhibits the growth and induces intracellular ROS generation in human colon cancer cells.** (**A**–**C**) SW620, HCT116, and HT-29 cells were treated with AS (0–300 μg/mL) for 24 h. Cell viability was determined using the MTT assay. (**D**, **E**) Cells were treated with 200 μg/mL AS for 0–120 min. 10 μM of DCFH_2_-DA was mixed in the culture medium 30 min before of each experiment and then intracellular ROS levels were measured and expressed in graph as a fold of the control. Each value is expressed as the mean ± SD (*n* = 3) and significant at ^*^p < 0.05; ^**^p < 0.01; and ^***^p < 0.001 when compared with control.

Initially, the cytotoxic effects of a fermented culture broth of *Antrodia salmonea* (AS) against human colon cancer cells; SW620, HCT116, and HT-29 was assessed. 0–300 μg/mL of AS was used to treat colon cancer cells for 24 h, and the cell viability was measured by MTT assay. AS treatment revealed its potential cytotoxic effects for SW620, HCT116, and HT-29 cells with IC_50_ values of 170, 163, and 235 μg/mL, respectively (Figure 1A–1C). The preliminary findings showed the evidence that AS treatment reduced the rate of proliferation and activated cell death for SW620, HCT116, and HT-29 cells. The relation of AS with ROS production in SW620 cells was measured by using a DCFH_2_-DA fluorescent probe under fluorescence microscopy. 200 μg/mL AS treated cells revealed utmost ROS generation at 90 min post AS treatment (Figure 1D and 1E).

### AS induces apoptosis in SW620 cells

**Figure 2:**
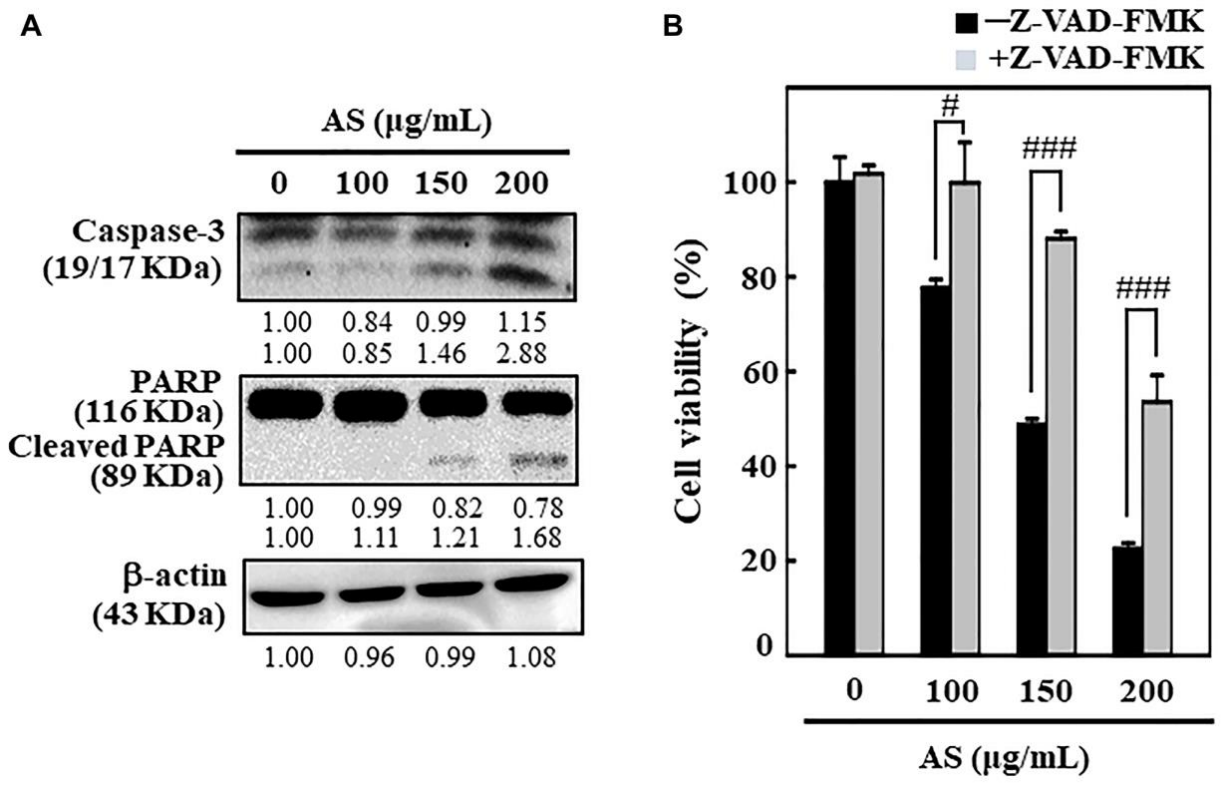
**AS induces apoptosis in colon cancer SW620 cells.** The cells were treated with 100, 150, and 200 μg/mL of AS for 24 h. (**A**) Caspase-3 and PARP protein levels were inspected by Western blotting. Relative changes in protein bands were analyzed by commercially available quantitative software (AlphaEase, Genetic Technology Inc. Miami, FL, USA), with control as 1-fold. (**B**) Cells treatment was done with 20 μM Z-VAD-FMK, a caspase inhibitor, for 1 h then followed by 100, 150, and 200 μg/mL AS for 24 h. MTT assay was performed to assess cell viability. The data are expressed as the mean ± SD (*n* = 3) of three replicates. The statistical significance was defined as ^#^*p* < 0.05 or ^###^*p* < 0.001 when compared with AS-treated cells.

To understand the mechanism behind AS-mediated cell death, SW620 cells were incubated with AS (0–200 μg/mL) for 24 h and the effects of AS on caspase-3 and PARP was examined. Western blot analysis showed that treatment of SW620 cells with AS (0–200 μg/mL) for 24 h released more active forms of caspase-3 (Figure 2A). Therapy with AS in SW620 cells significantly rose the expression level of active caspase-3 relative to the dose. Furthermore, the degradation of PARP, which is a well-known apoptosis marker was also observed. As shown in Figure 2A, compared with control cells AS treatment substantially cleaved the PARP protein.

Interestingly, Z-VAD-FMK, a pan-caspase inhibitor, significantly reduced AS-induced cell death (Figure 2B), which additionally supported AS-induced apoptotic cell death. This evidence reveals that AS treatment triggered apoptosis in SW620 cells.

### AS induces mitochondria dysfunction and dysregulates the Bax and Bcl-2 ratio in SW620 cells

**Figure 3:**
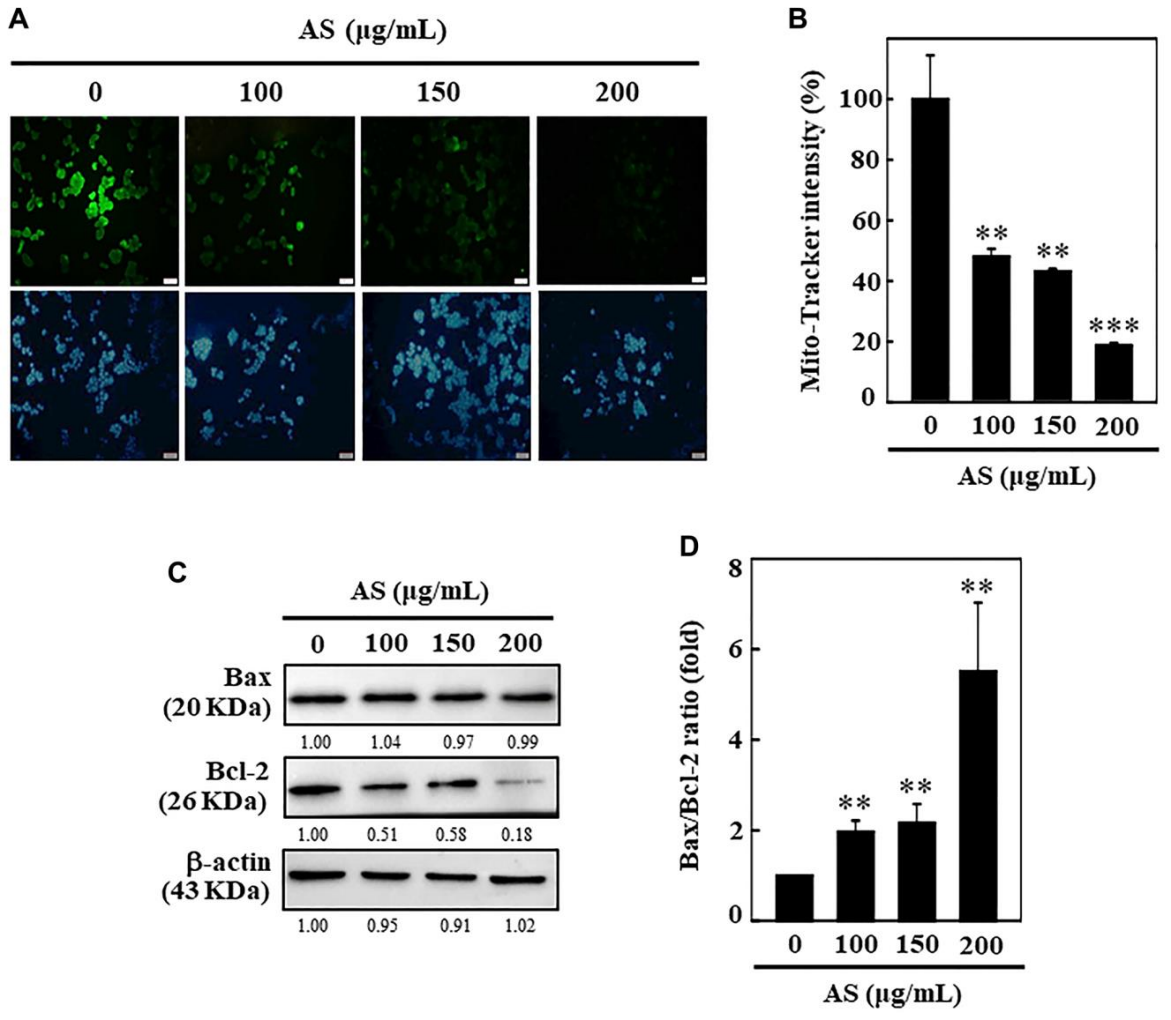
**AS induces mitochondrial dysfunction and Bax/Bcl-2 dysregulation in SW620 cells.** (**A**, **B**) Depolarization of mitochondrial membrane potential (ΔΨm) was measured using Mito-tracker green detection kit. 100, 150, and 200 μg/mL of AS was used to treat cells for 24 h. AS increases the ratio of Bax/Bcl-2. (**C**) Dose-dependent (100, 150, and 200 μg/mL) effects of AS on Bax and Bcl-2 proteins were estimated through Western blotting. (**D**) Relative changes in the ratio of Bax and Bcl-2 with the different doses of AS were estimated by commercially available quantitative software with control as 1-fold. Values are expressed as the mean ± SD (*n* = 3). Statistical significance was defined as ^**^p < 0.01 or ^***^p < 0.001 when compared with control.

To examine whether AS-induced apoptosis was regulated by the mitochondrial pathway, 0–200 μg/mL of AS was treated to SW620 cells for 24 h, and then the changes occurred in the mitochondrial membrane potential were observed by using Mito-Tracker assay kit. Mito-Tracker is a green fluorescent dye which has ability to stain the mitochondria in viable cells, and its aggregation depends upon the membrane potential [28]. Control cells exhibited bright green fluorescence, while the level of green fluorescence decreased in a dose-dependent manner when cells were exposed to AS for 24 h, specifying that AS treatment induced mitochondrial membrane permeability (Figure 3A and 3B). This data strongly suggests that mitochondrial function was critically diminished by AS-induced apoptosis in SW620 cells. Apoptosis due to mitochondrial damage, acting as apoptosis activators (Bax) or inhibitors (Bcl-2), has been reported to be controlled by the Bcl-2 family proteins [29]. The proportion between Bax and Bcl-2 has crucial role in balancing homeostasis with regard to apoptosis. The results showed that treatment with AS for SW620 cells did not induce pro-apoptotic Bax expression and further reduced anti-apoptotic protein Bcl-2 suggesting that AS could alter the ratio of Bax and Bcl-2 and thus activated apoptosis (Figure 3C and 3D). These results suggest that AS treatment effectively promotes apoptosis mediated by mitochondrial pathway in SW620 cells.

### AS activates autophagy by increasing AVOs formation and LC3 accumulation in SW620 cells

**Figure 4:**
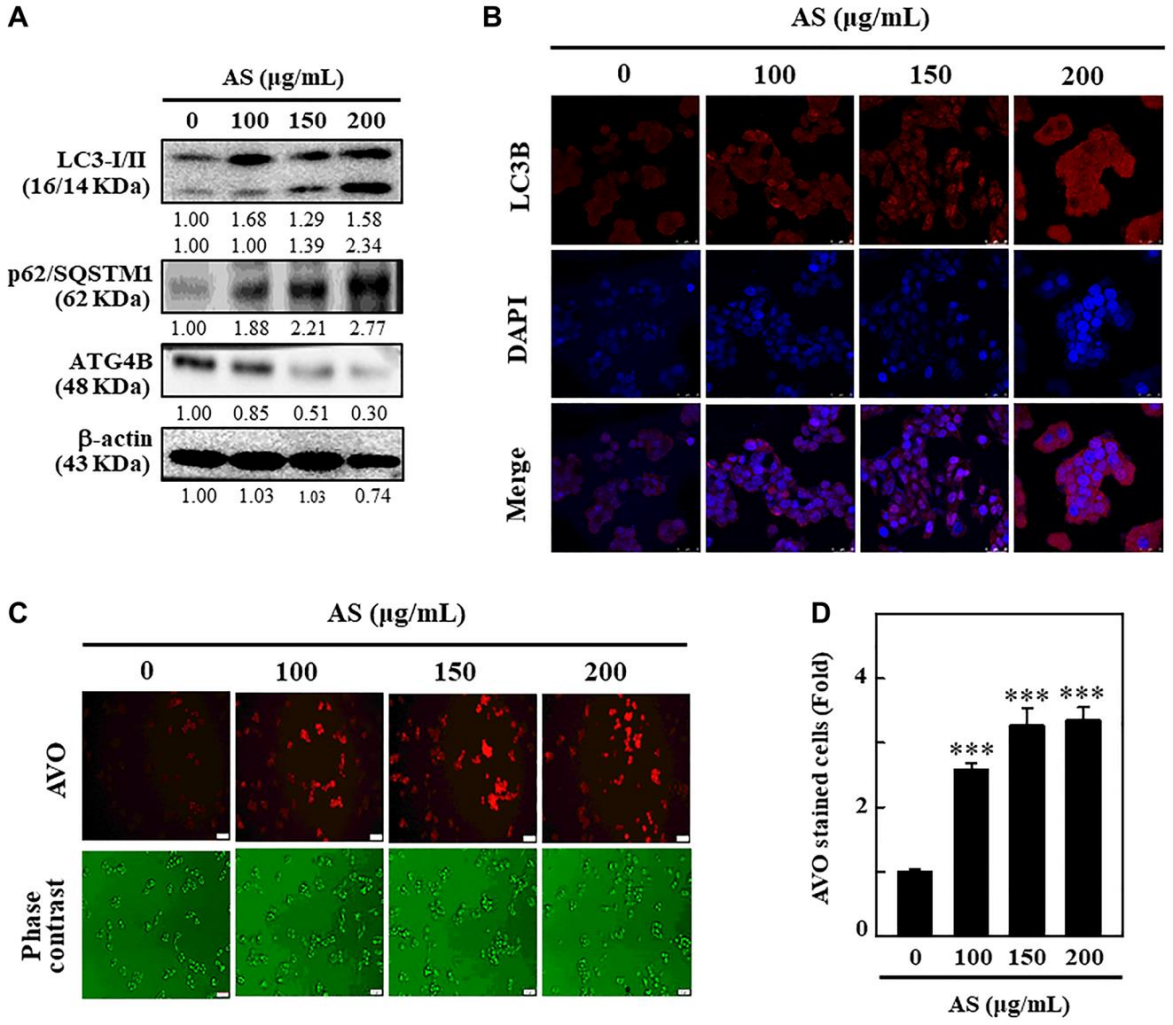
**Induction of autophagy in SW620 cells by AS treatment.** (**A**) AS triggers autophagy signaling molecules in SW620 cells. Cells were treated with AS (100, 150, and 200 μg/mL for 24 h, and then conversion of LC3-I to LC3-II and the expressions of p62/SQSTM1 and ATG4B were determined by Western blotting. β-actin was used as a loading control. Relative changes in the intensities of protein bands were measured by commercially available quantitative software. (**B**) Immunofluorescence detection of LC3B in the cells treated with AS (100, 150, and 200 μg/mL) for 24 h. (**C**) Cells were treated with 100, 150, and 200 μg/mL of AS for 24 h and stained with acridine orange for AVOs detection in untreated or AS-treated cells. The cells were examined through a red filter of fluorescence microscope. (**D**) The fold of cells with AVOs are represented in bar diagram. Values are expressed as the mean ± SD (*n* = 3). ^***^p < 0.001 is significant when compared with untreated control cells.

LC3 being a promising marker for autophagy, its distribution in intracellular region [30], has been examined to find out whether AS can induce autophagy in SW620 cells. The cells treated with 0-200 μg/mL AS for 24 h were subjected to Western blot to understand the effects of AS on the distribution of LC3-I and LC3-II. The outcomes revealed that the accumulation of LC3-I and LC3-II was dose-dependently increased following AS treatment. The high dose (200 μg/mL) of AS notably increased the high accumulation of LC3-II (Figure 4A).

p62 or sequestosome 1 (SQSTM1), is an important protein that binds directly to LC3 and then undergoes self-degradation while autophagy occurs [31]. Being a multifunctional ubiquitin-binding protein, p62/SQSTM1 involves in many important processes of autophagy [32]. However, p62/SQSTM1 expression level increases with impaired autophagy [33]. From the results, it was known that the level of expression of p62/SQSTM1 remarkably increased with AS incubation after 24 h in a dose-dependent fashion suggesting that AS-induced autophagy in SW620 cells. The increase in p62/SQSTM1 levels was linked with the increasing accumulation of LC3 in SW620 cells (Figure 4A).

To confirm the role of ROS on ATG4B regulated autophagy activation, the level of expression of ATG4B protein was examined in SW620 cells after treatment with AS. Furthermore, dose-dependent treatment of AS notably decreased ATG4B expression in SW620 cells in comparison to control cells (Figure 4A). These findings explain new insights into the crucial role of AS-induced ROS generation in the down-regulation of ATG4B level in SW620 cells. These findings clearly indicated that after AS (100, 150, and 200 μg/mL) therapy, LC3-II accumulation occurred, giving complete evidence for the induction of autophagy by AS in SW620 cells (Figure 4B). The formation of AVOs is a typical aspect of autophagy, so the sequential impact of AS on AVOs formation was monitored by using AO staining and fluorescence microscopy. Dose-dependent treatment of AS increased the formation of AVOs with maximum expression at 200 μg/mL revealing that AS could induce autophagic flux in SW620 cells (Figure 4C and 4D).

### AS activates autophagy signaling cascades as a survival mechanism in SW620 cells

**Figure 5:**
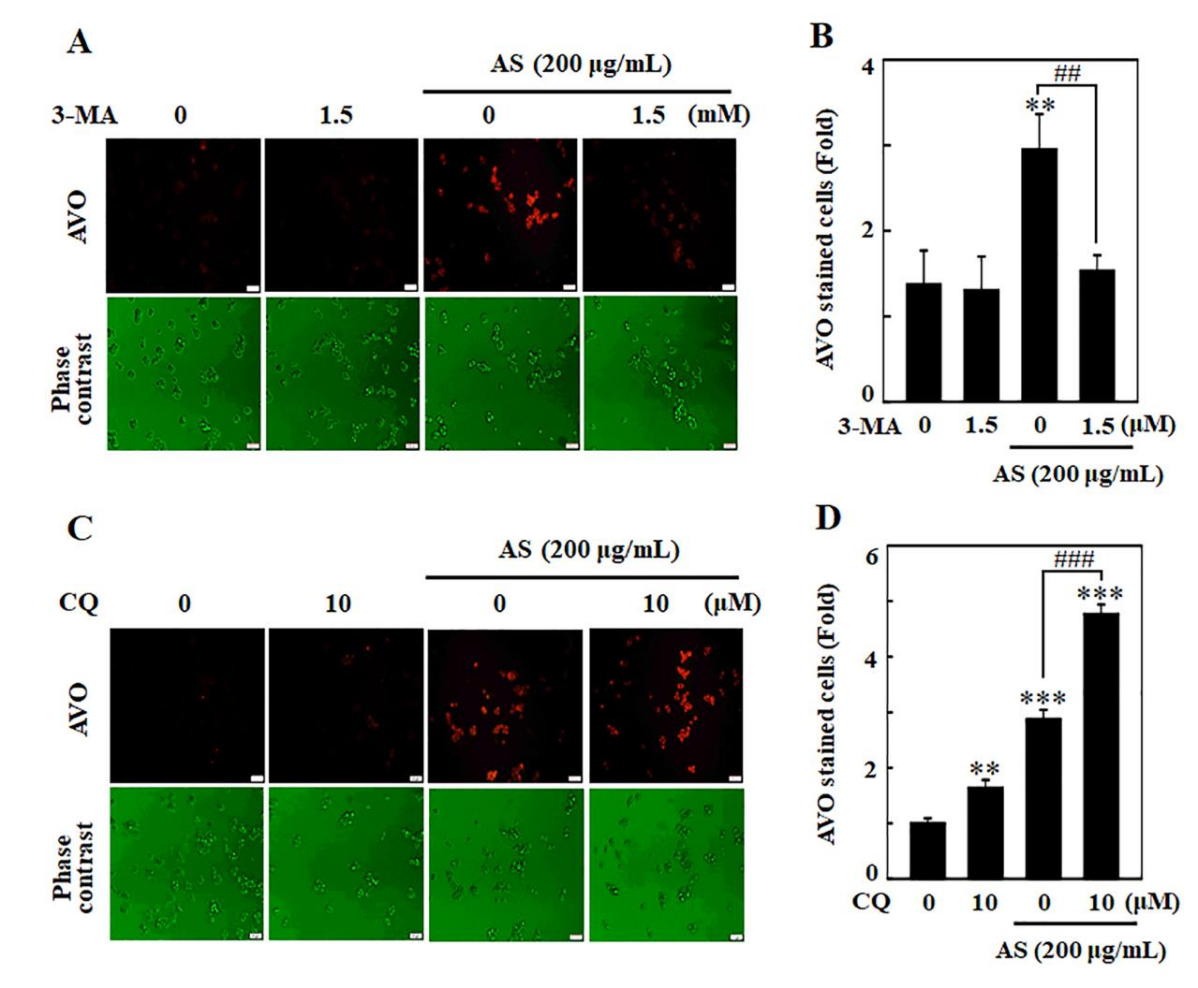
**Inhibition of AS-induced autophagy by 3-MA and CQ inhibitors in SW620 cells**. Cells were pretreated with autophagy inhibitor (**A**) 3-MA (1.5 mM) for 1 h followed by incubation with or without AS (200 μg/mL) for 24 h. Cells were stained with acridine orange and visualized under a red filter fluorescence microscope for AVOs measurement and (**B**) the fold of cells with AVOs are represented in bar diagram. Cells were pretreated with autophagy inhibitor (**C**) CQ (10 μM) for 1 h followed by incubation with or without AS (200 μg/mL) for 24 h. Cells were stained with acridine orange and visualized under a red filter fluorescence microscope for AVOs measurement and (**D**) the fold of cells with AVOs are represented in bar diagram. Results are expressed as the mean ± SD of three independent assays. ^**^p < 0.01 or ^***^p < 0.001 is significant when compared with untreated control cells as well as ^##^*p* < 0.01 or ^###^*p* < 0.001 is significant when compared with AS-treated cells.

Existing evidence suggests contradiction on role of autophagy whether it controls cell death or survival in response to different stimuli. The role of AS-induced autophagy in SW620 cells has therefore been investigated by blocking autophagy using pharmacological inhibitors like 3-MA and CQ. 3-MA and CQ were used to interrupt lysosomal function and avoid early and late autophagy process. To accomplish this objective, cells were handled with 3-MA/CQ, AS alone or in combination. The results depicted that 3-MA (1.5 mM) halted AS-induced AVOs formation, indicating that in an early stage of autophagy there was inhibition of LC3-II accumulation (Figure 5A and 5B). Compared to AS treatment alone, 10 μM CQ treated cells resulted in vast emergence of AVOs, suggesting stimulation of LC3-II accumulation at the late stage of autophagy (Figure 5C and 5D).

### AS dysregulates ratio of Beclin-1/Bcl-2

**Figure 6:**
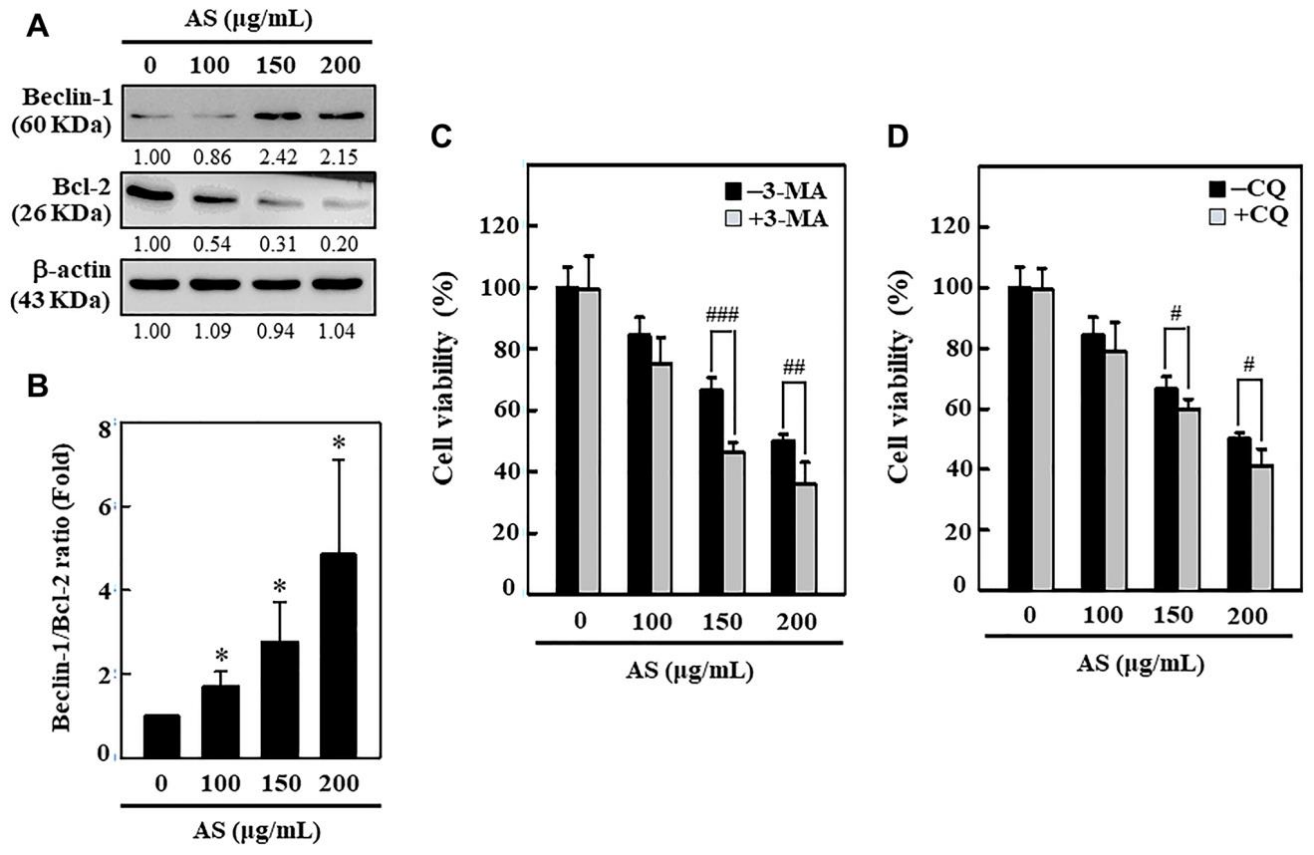
**Induction of autophagy in SW620 cells by AS treatment.** (**A**) AS increases the ratio of Beclin-1/Bcl-2. Dose-dependent AS (100, 150, and 200 μg/mL) effects of AS on changes in Beclin-1 and Bcl-2 proteins were determined by Western blotting. (**B**) Relative changes in the ratio of Beclin1/Bcl-2 in accord with the dose were measured by commercially available quantitative software with the control representing 1-fold. Enhancement of AS-induced cell death by 3-MA and CQ inhibitors in SW620 cells. Cells were first treated with autophagy inhibitors (**C**) 3-MA (1.5 mM) and (**D**) CQ (10 μM) for 1 h and then incubated in presence or absence of AS (0-200 μg/mL) for 24 h. Cell viability was analyzed by the MTT assay. Results are expressed as the mean ± SD of three independent assays. ^*^p < 0.05 is significant when compared with untreated control cells and ^#^*p* < 0.05; ^##^*p* < 0.01; ^###^*p* < 0.001 is significant when compared with AS-treated cells.

Bcl-2 family proteins serve as crucial key controllers of mitochondrial-mediated apoptosis and function as either activators or inhibitors [34]. The interplay between Beclin-1 (autophagy protein) and Bcl-2 has been shown to be complex and Bcl-2 can reduce the Beclin-1 pro-autophagy property [35]. Hence, the effect of AS on Bcl-2 protein and its function in Beclin-1 (pro-autophagic) expression in SW620 cells was studied. Western blotting data revealed that Beclin-1 proteins dramatically increased with AS in dose-dependent manner (0–200 μg/mL, 24 h) in SW620 cells (Figure 6A). Furthermore, the expression of Bcl-2 was down-regulated with AS in dose-dependent manner (Figure 6A). The quantified ratio of Beclin-1/Bcl-2 was strongly increased by AS (Figure 6B), suggesting an induction of autophagy in SW620 cells. Pretreatment of cells with 3-MA (1.5 mM) and CQ (10 μM) successfully improved AS-induced cell death, as shown in Figure 6C and 6D. These findings indicated that autophagy was caused by AS as a mechanism of survival in SW620cells.

### Inhibition of cytoprotective autophagy enhances AS-induced apoptosis in SW620 cells

**Figure 7:**
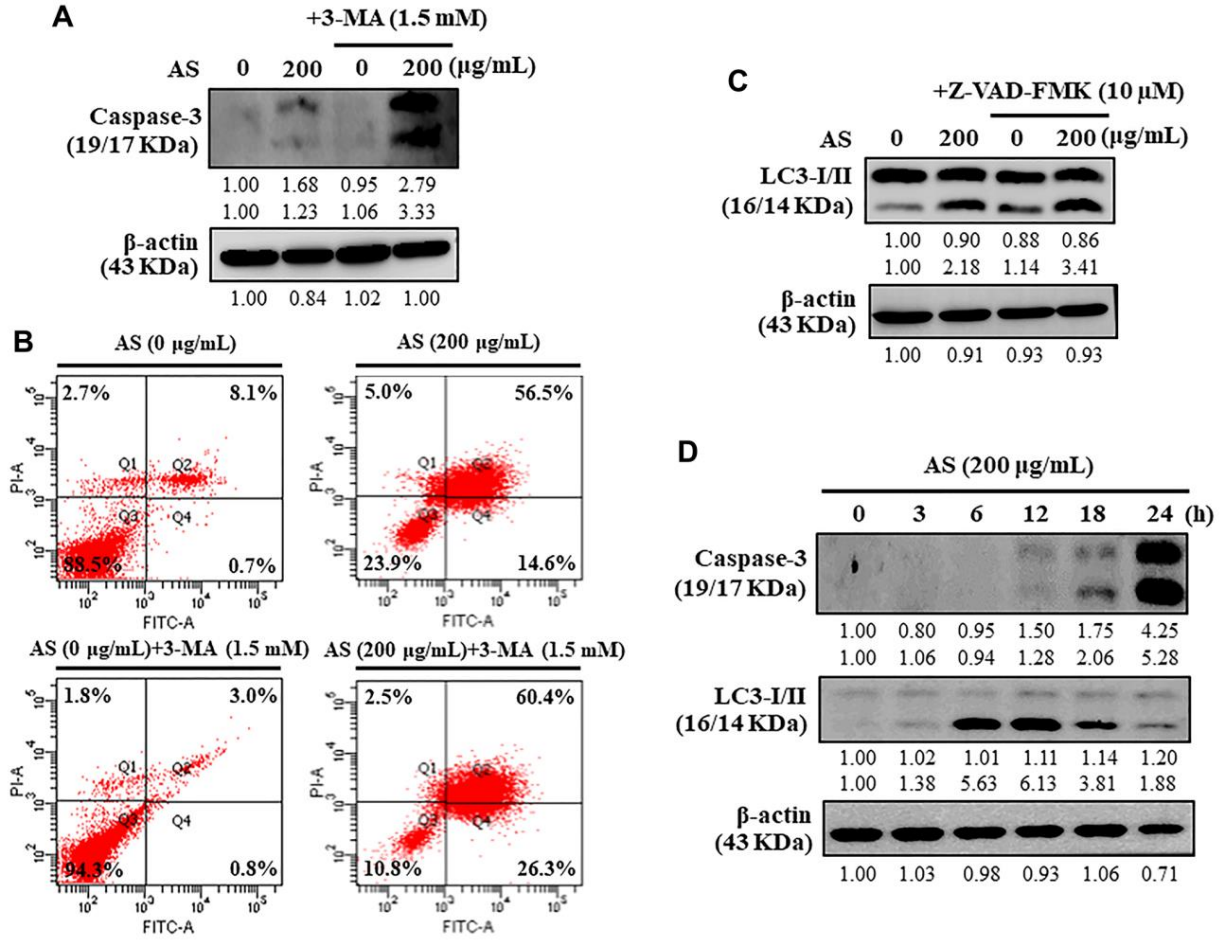
**Interplay between AS-induced autophagy and apoptosis in SW620 cells.** (**A**) At first the cells were treated with or without 3-MA (1.5 mM) for 1 h, and incubated with AS (200 μg/mL) for 24 h. Caspase-3 cleavage was determined by Western blotting. (**B**) AnnexinV-FITC/PI staining was carried out to know about early/late apoptosis or necrosis. Flow cytometry analysis with or without 3-MA (1.5 mM) for 1 h, and incubated with AS (200 μg/mL) for 24 h. (**C**) The cells were pretreated with or without Z-VAD-FMK (10 μM) for 1 h, followed by incubation with AS (200 μg/mL) for 24 h. (**D**) Time-dependent AS (200 μg/mL for 0–24 h) effects of AS on Caspase-3 and LC3-I/II proteins were estimated by Western blotting. Relative changes in accord with the time were determined by commercially available quantitative software representing the control as 1-fold.

Under different conditions, autophagy can proceed to activate caspase-dependent apoptosis [36]. Apoptosis was examined to investigate the intervention of AS in the relationship between apoptosis and autophagy by treating the cells with 3-MA (1.5 mM), autophagy inhibitor. 3-MA treated SW620 cells resulted in enhancement of AS-induced caspase-3 activation (Figure 7A). To know if AS induces apoptosis (early/late) or necrosis in SW620 cells, the Annexin V-FITC and PI assay was performed. Annexin V-FITC particularly stains phosphatidylserine whereas PI stains DNA residues only [37]. The results from flow cytometry exhibited that treatment of SW620 cells with AS for 24 h showed early apoptotic cells represented in Q4 were 0.7% and 14.6% with 0 and 200 μg/mL of AS treatment respectively and late apoptotic cells represented in Q2 were 8.1% and 56.5% with 0 and 200 μg/mL of AS treatment respectively (Figure 7B). Additionally, when flow cytometry analysis was carried out on cells treated with 3-MA (1.5 mM for 1 h) and AS (200 μg/mL for 24 h), it showed that AS-mediated autophagy inhibition increased the occurrence of apoptotic cell death mediated by AS. (Figure 7B).

### Inhibition of apoptosis does not affect AS-induced autophagy in SW620 cells

Caspases normally remains in an inactive form while their activation take crucial part in the apoptosis [38]. Cells were treated with an apoptosis inhibitor (Z-VAD-FMK) to resolve the intrusion of AS with the interlinkage between apoptosis and autophagy, and improvements in the co-localization of LC3-II accumulation were determined by Western blot. Interestingly, Z-VAD-FMK (10 μM) inhibition of caspase-3 activation had no effects on LC3-II accumulation against AS-induced apoptosis in SW620 cells (Figure 7C). Hence, the inhibitor study findings revealed that inhibition of apoptosis via Z-VAD-FMK did not affect AS-induced autophagy.

### AS induces autophagy and then apoptosis

Western blot data revealed that treatment with AS increased activation of caspase-3 in a time-dependent (0–24 h) manner showing maximum activation at 24 h (Figure 7D). In addition, the time-dependent intracellular transformation of LC3-I to LC3-II was increased with AS treatment and maximum accumulation at 12 h (Figure 7D) suggesting that AS induced cytoprotective autophagy and then apoptosis.

### ROS involves in AS-induced autophagy and apoptosis in SW620 cells

**Figure 8:**
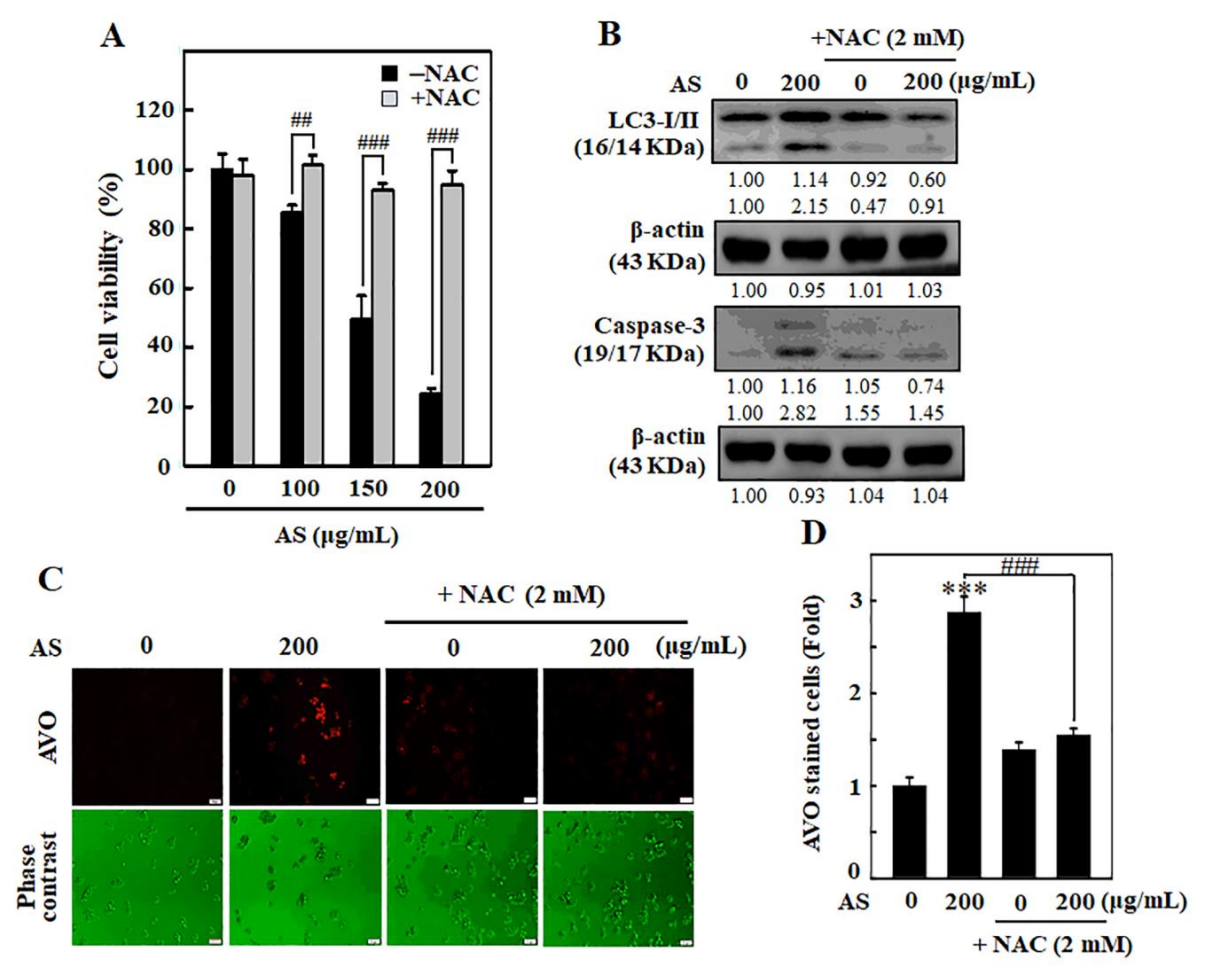
**ROS is involved in AS-activated autophagy and apoptosis in SW620 cells.** Cells were earlier treated with or without ROS inhibitor (NAC, 1 mM) for 1 h, followed by AS (100–200 μg/mL) treatment for 24 h. (**A**) Cell viability was assayed by the MTT assay. (**B**) The expressions of LC3-I/II and Caspase-3 were measured by Western blotting. The relative changes in the expression of protein bands were estimated by commercially available quantitative software representing control as 1-fold. (**C**) Cells were stained with acridine orange and visualized through a red filter fluorescence microscope to detect AVOs. (**D**) The fold of cells with AVOs are represented in bar diagram. Results are expressed as the mean ± SD of three independent assays. ^***^p < 0.001 is significant when compared with untreated control cells and ^##^*p* < 0.01 or ^###^*p* < 0.001 is significant when compared with AS-treated cells.

To examine whether AS-induced apoptosis/autophagy is dependent with ROS production, SW620 cells were incubated along with ROS inhibitor (NAC, 1 mM) for 1 h earlier than AS treatment (0–200 μg/mL). After that MTT assay was done to know the cell viability. Remarkably, cells pre-treated with NAC significantly rescued the AS-induced cell death (Figure 8A). Western blotting results showed that pre-incubation of cells with NAC had inhibitory effects on AS-induced LC3-I/II accumulation and caspase-3 activation (Figure 8B). To investigate the importance of ROS production in AS-induced autophagy, AO staining was carried out. Cells treated with AS induced AVOs formation, whereas NAC pre-treated cells showed significantly reduced AVOs formation in SW620 cells (Figure 8C and 8D). These findings confirm that AS induced ROS-regulated autophagy and apoptosis in SW620 cells.

### AS upregulates the ERK signaling pathways in SW620 cells

**Figure 9:**
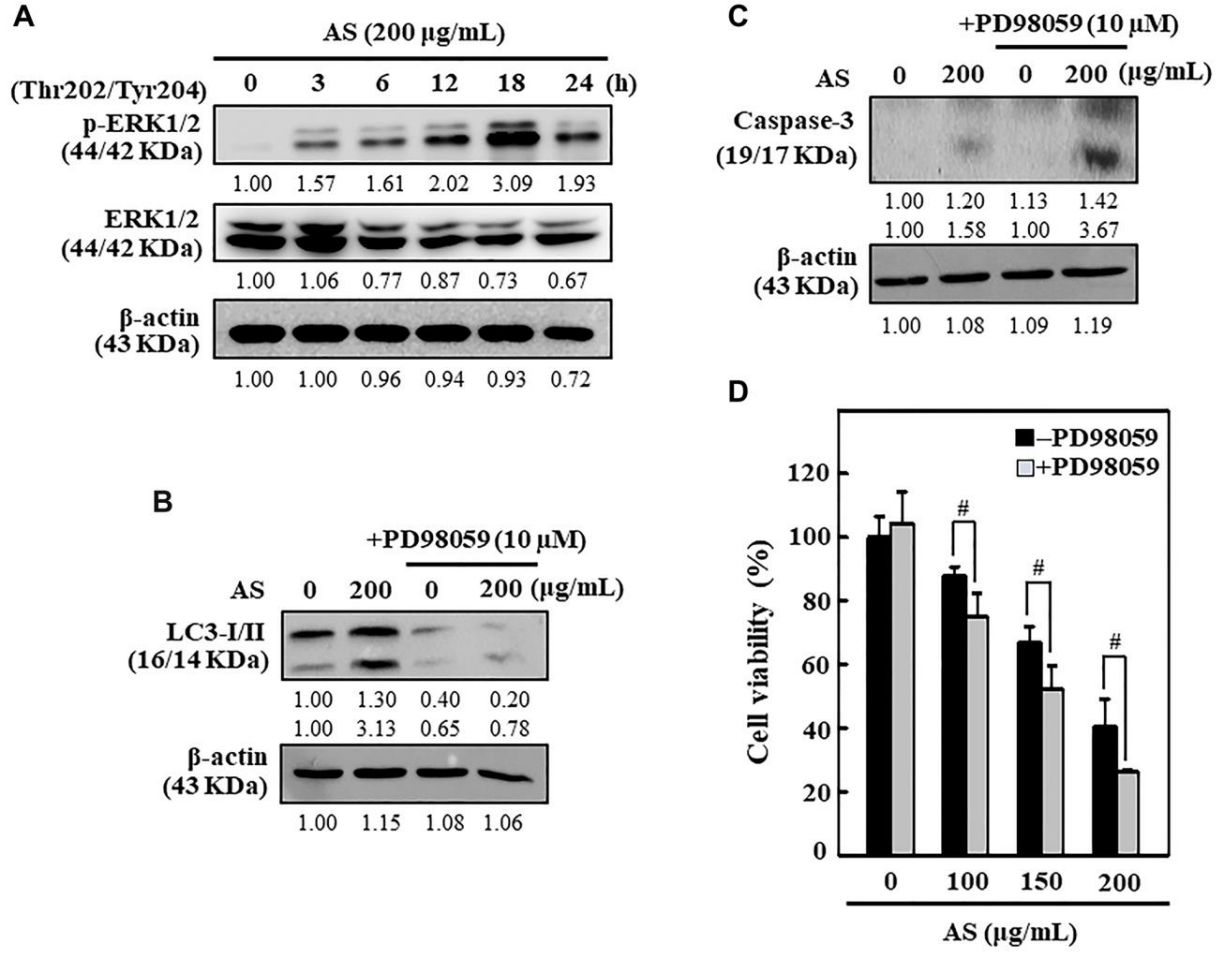
**ERK activation inhibited apoptosis through enhanced cytoprotective autophagy in AS-treated SW620 cells.** (**A**) Cells were treated with AS (200 μg/mL) for 0–24 h and immunoblotting was performed to measure the levels of p(Thr202/Tyr204)-ERK1/2 and ERK1/2. Cells were pretreated with or without ERK inhibitor (PD98059, 10 μM) for 1 h, followed by 200 μg/mL AS treatment for 24 h. (**B**) The expressions of LC3-I/II and (**C**) caspase-3 were assessed by Western blotting. Relative changes in the expression of protein bands were measured by commercially available quantitative software with the control representing as 1-fold. (**D**) Cell viability was measured by the MTT assay. Earlier the cells were treated with or without ERK inhibitor (PD98059, 10 μM) for 1 h, and then followed by AS (100, 150, and 200 μg/mL) treatment for 24 h. Results are expressed as the mean ± SD of three independent assays. ^#^*p* < 0.05 is significant when compared with AS-treated cells.

The ERK pathway has chief role in controlling cell growth, survival, and differentiation and decides the cellular fate against external stimuli [39]. The ERK expression and phosphorylation patterns due to the effect of AS was tested in SW620 cells. Western blot data revealed that AS activated the p-ERK in SW620 cells in time-dependent manner (Figure 9A). The effects of ERK inhibition on caspase-3 activation, LC3-I/II accumulation, and cell viability was further examined. As displayed in Figure 9B and 9C, ERK inhibitor PD98059 pretreatment along with AS for SW620 cells significantly decreased the LC3-I/II accumulation but in contrast enhanced the caspase-3 activation. Apart from this, cell viability data too indicated that SW620 cells treated with both AS and PD98059 showed remarkable suppression in cell viability in comparison to the AS alone treated cells (Figure 9D). Hence, these observations decided that the activation of ERK is crucial in AS-mediated cytoprotective autophagy and apoptosis.

### AS inhibits AKT/mTOR pathway and activates cytoprotective autophagy in SW620 cells

**Figure 10:**
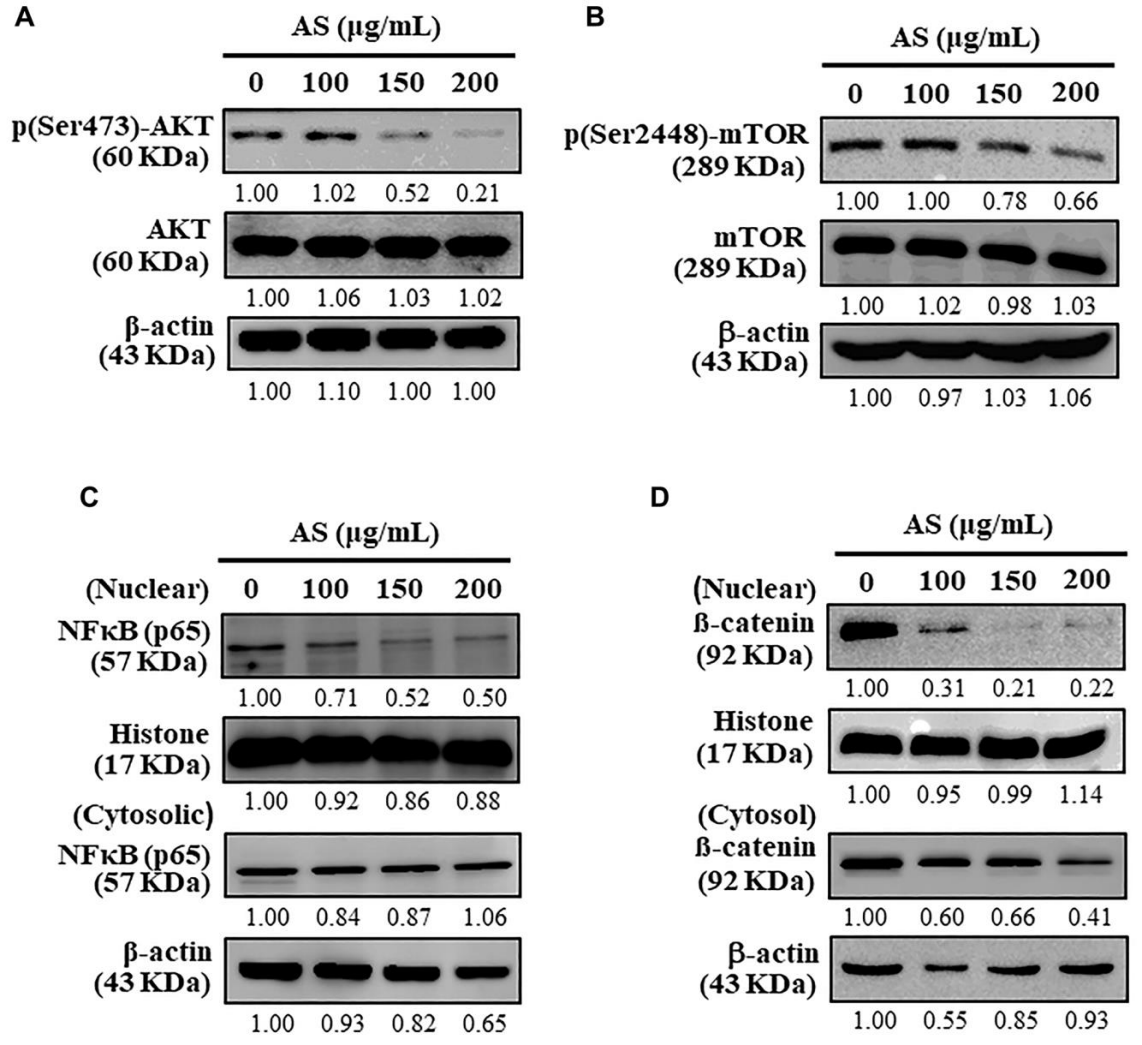
**Inhibitory effects of AS on AKT, mTOR, and NFκB signaling pathways in SW620 cells.** (**A**) Cells were treated with AS (100, 150, and 200 μg/mL) for 24 h. Immunoblotting was performed to measure the levels of p(Ser473)-AKT and AKT. (**B**) Cells were treated with AS (100, 150, and 200 μg/mL) for 24 h. Immunoblotting was performed to measure the levels of p(Ser2448)-mTOR and mTOR. (**C**) Cells were treated with AS (100, 150, and 200 μg/mL) for 24 h. Immunoblotting was performed to measure the levels of nuclear and cytosolic NFκB (p65). (**D**) Cells were treated with AS (100, 150, and 200 μg/mL) for 24 h. Immunoblotting was performed to measure the levels of nuclear and cytosolic β-catenin. Relative changes in the intensities of protein bands were measured using commercially available quantitative software with the control representing as 1-fold.

Autophagy is a complex cellular self-catabolic mechanism that is closely regulated by upstream modulators, mainly the AKT and mTOR signalling pathway [40]. The effects of AS on downstream proteins, including AKT and mTOR was determined using Western blot analysis which showed that AS treatment notably attenuated the phosphorylation of AKT as well as mTOR in a dose-dependent fashion in SW620 cells (Figure 10A and 10B). Further, AS treatment significantly inhibited nuclear NFκB levels (Figure 10C) and β-catenin levels (Figure 10D). These findings revealed AS suppressed AKT/mTOR signaling cascades, NFκB, and β-catenin expression which in turn activated cytoprotective autophagy in human colon SW620 cells.

### Time-dependent effects of AS on body weight of AOM/DSS-treated ICR mice

**Figure 11:**
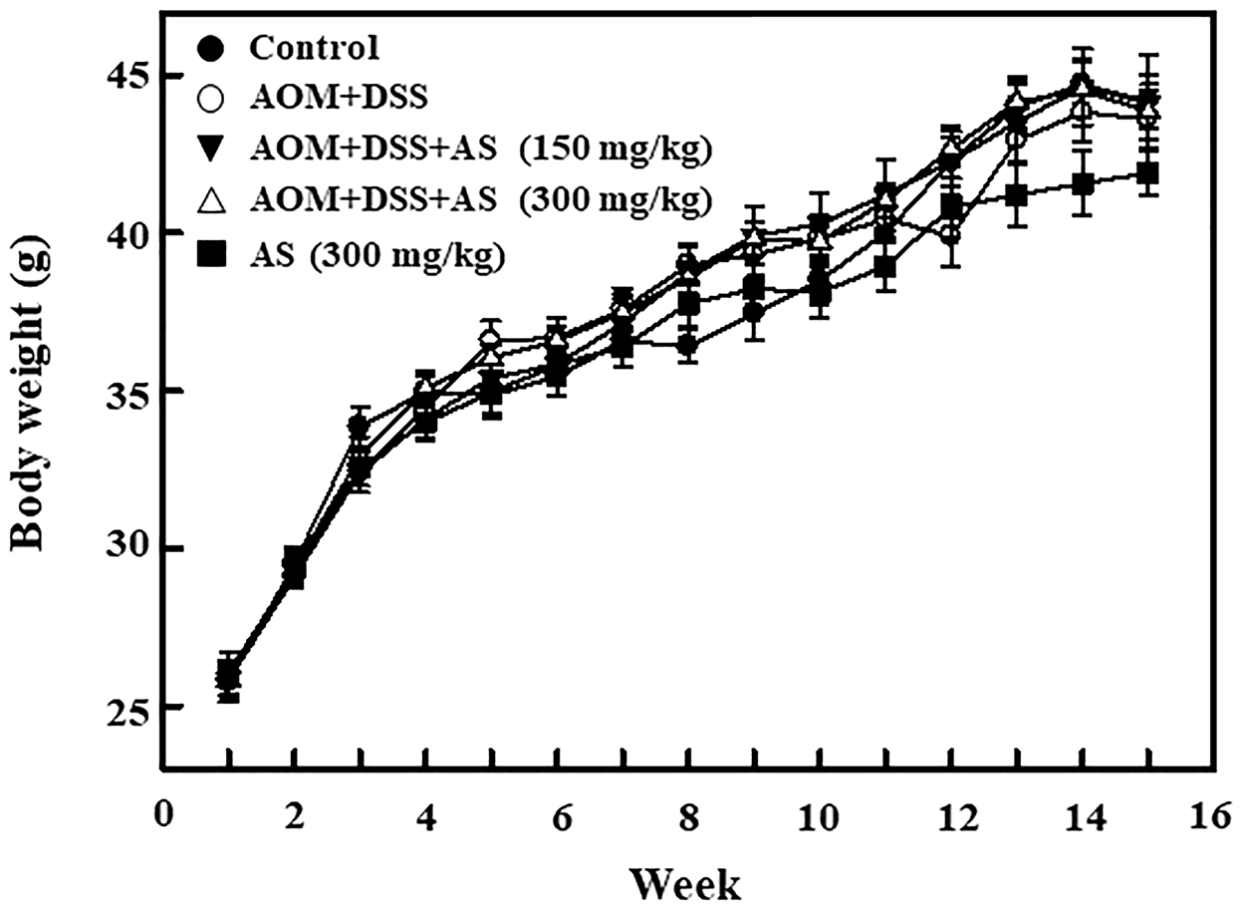
**Time-dependent effects of AS on body weight of AOM/DSS-treated ICR mice.** Mice were injected intraperitoneally with a single dose (15 mg/kg) of AOM (the first week) followed by 3 times of 2% DSS given in the drinking water every two days (the second week). AS (150 and 300 mg/kg) was given oral administration every two days and sacrificed on 15 weeks after CAC induction. Body weight was recorded for every week and the values were expressed as mean ± SEM (*n* = 9~13) mice/group. Statistical significance was defined as ^*^p < 0.05; ^**^p < 0.01; ^***^p < 0.001 compared with control. ^#^*p* < 0.05; ^##^*p* < 0.01; ^###^*p* < 0.001 compared with AOM/DSS-treated cells.

In order to induce CAC, in first week 15 mg/kg of AOM was provided for the group II mice by intraperitoneal injection and in second week followed by 2% DSS for three times along with drinking water every two days. On 15 weeks of CAC induction the mice were sacrificed. The body weight was recorded every week for monitoring the effect of CAC induction. The effects of AS in body weight was monitored in AOM/DSS-induced mice. Furthermore, the increase of body weight was time-dependently alleviated by the combination of AS and AOM/DSS as well as AS alone treatment (Figure 11). The details about changes in the weight of liver, kidney, and spleen are presented in Table 1. There were no any significant changes observed after exposure to 150 and 300 mg/kg of AS suggesting that there were no signs of significant toxicity and AS showed no side effects when treated with this dose.

**Table 1:** The effects of AS on the weight of liver, and spleen on AOM/DSS-treated ICR mice.

**Treatment**	**Liver (g)**	**Kidney (g)**	**Spleen (g)**
Control	2.25 ± 0.22	0.78 ± 0.04	0.17 ± 0.01
AOM (15 mg/kg) + DSS (2%)	2.39 ± 0.10	0.75 ± 0.03	0.16 ± 0.02
+ AS (150 mg/kg)	2.15 ± 0.05	0.69 ± 0.02	0.12 ± 0.01
+ AS (300 mg/kg)	2.22 ± 0.06	0.74 ± 0.03	0.13 ± 0.01
+ AS (300 mg/kg)	2.21 ± 0.08	0.73 ± 0.03	0.15 ± 0.01

### AS decreases colonic weight and increases colonic shortening of AOM/DSS-induced ICR mice

**Figure 12:**
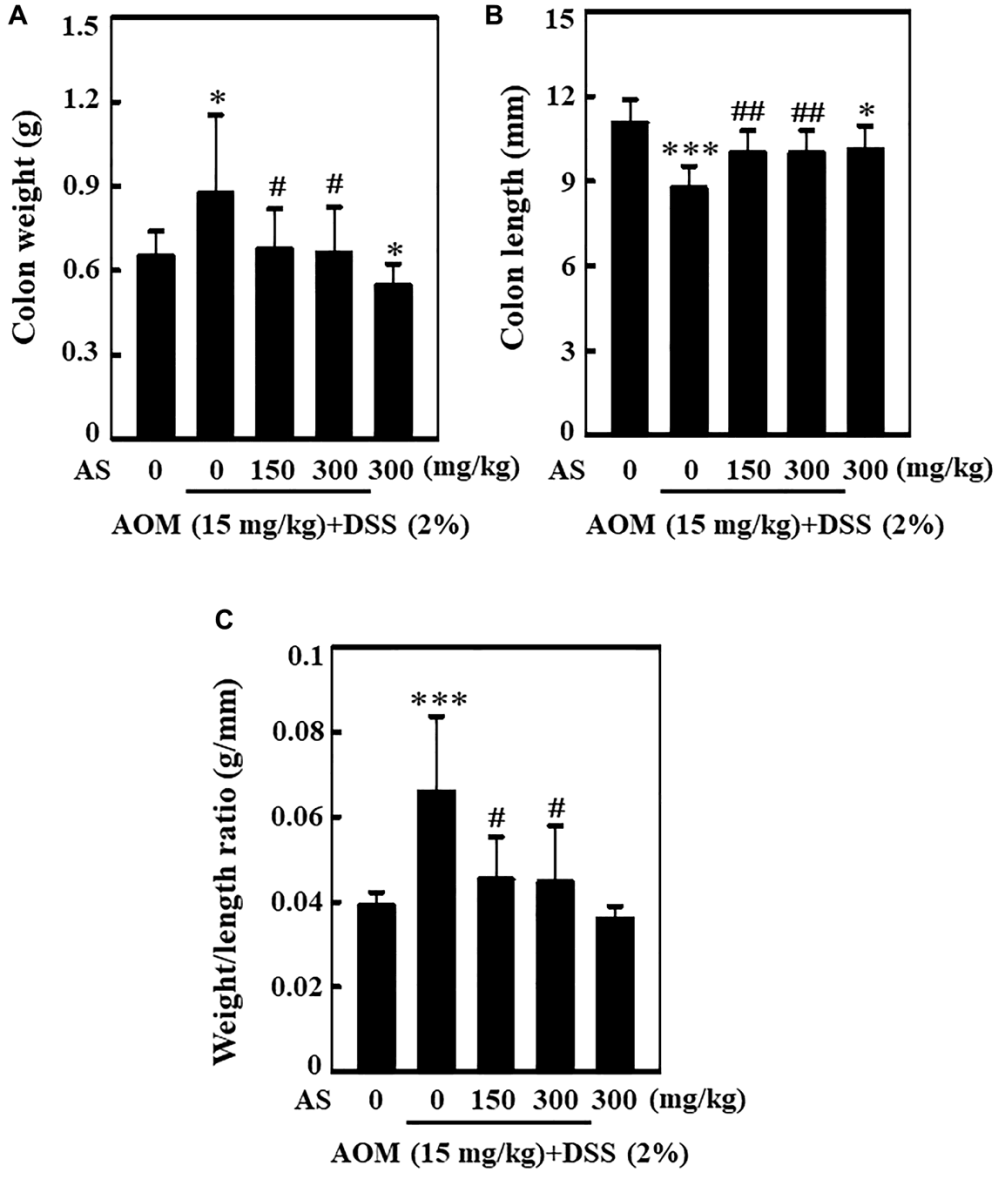
**AS decreased AOM/DSS-induced colonic weight and increased AOM/DSS-induced colonic shortening of ICR mice.** Mice were injected intraperitoneally with a single dose (15 mg/kg) of AOM (the first week) followed by 3 times of 2% DSS given in the drinking water every two days (the second week). AS (150 and 300 mg/kg) was given oral administration every two days. Mice were sacrificed on 15 weeks after CAC induction. (**A**) The entire colon was removed and the colon weight (g) and (**B**) length (mm) were measured. (**C**) Effect of AS in colon weight to colon length ratio. Statistical significance was defined as ^*^p < 0.05 or ^***^p < 0.001 compared with control. ^#^*p* < 0.05 or ^##^*p* < 0.01 compared with AOM/DSS-treated cells.

Single dose of 15 mg/kg of AOM was administered to the mice in first week with intraperitoneal injection followed by three times of 2% DSS in second week given by mixing with drinking water every two days. Post AOM/DSS induction, 150 and 300 mg/kg AS was given orally for every two days and the mice were sacrificed on 15 weeks of CAC induction. The entire colon was separated from the body and the weight as well as length was measured. Similarly, ratio of colon weight and length was calculated. While comparing with mice treated with control, AOM/DSS induction could increase the weight of colon and decrease the length of colon (Figure 12A and 12B). However, this increase in weight of colon was found to be reduced by the treatment of AS in comparison to other group (Figure 12A). Likewise, when treated with 300 mg/kg of AS the decrease in colon length was relieved (Figure 12B). Similarly increase in the ratio of colon weight to length might be due to the consequence of thickening of mucosa but AS treatment remarkably decreased this ratio indicating that AS had played important role in the reduction of AOM/DSS-induced CAC (Figure 12C).

### AS prevents AOM/DSS-induced tumor formation in ICR mice

**Figure 13:**
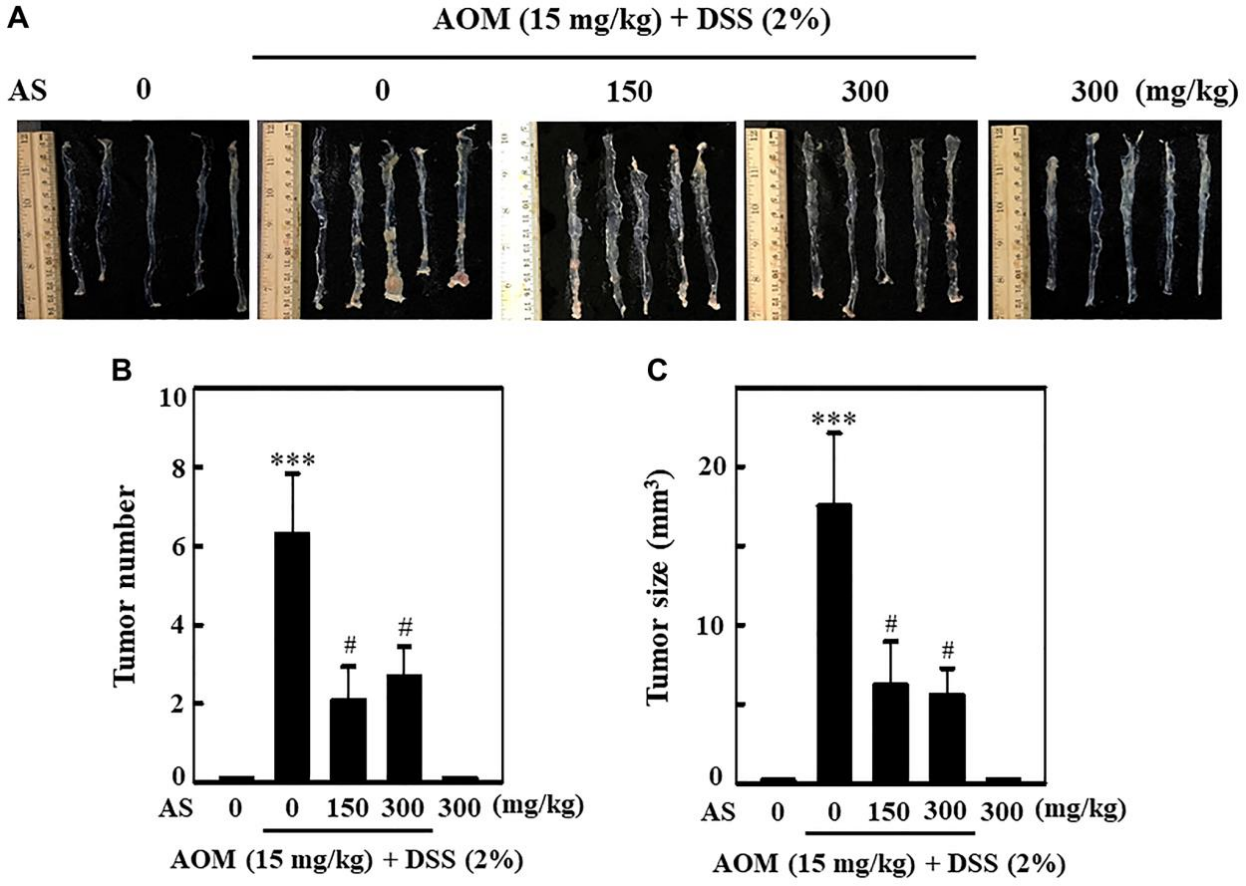
**AS prevented AOM/DSS-induced tumor formation in ICR mice.** Mice were injected intraperitoneally with a single dose (15 mg/kg) of AOM (the first week) followed by 3 times of 2% DSS given in the drinking water every two days (the second week). AS (150 and 300 mg/kg) was given oral administration every two days. Mice were sacrificed on 15 weeks after CAC induction. (**A**) The colon was removed and photographed. (**B**) Tumor numbers and (**C**) size were counted and calculated. Statistical significance was defined as ^***^p < 0.001 compared with control or ^#^*p* < 0.05 compared with AOM/DSS-treated cells.

To know the effect of AS in the formation of tumor in colon, in first week a single dose of 15 mg/kg of AOM was injected into mice intraperitoneally and in second week followed by three times of 2% DSS given with drinking water every two days. Likewise, 150 and 300 mg/kg AS was administrated orally and the mice were sacrificed on week 15. At the end of the experiment, the colon was removed, photographed (Figure 13A) and tumor formation was analyzed. The results disclosed that without AS treatment, AOM/DSS-induced mice showed notable increase in number and size of tumor in the colons, but along with AS treatment the number and size of tumor was markedly reduced in AOM/DSS-induced mice (Figure 13B and 13C).

### AS inhibits progression of CAC in AOM/DSS-treated ICR mice

**Figure 14:**
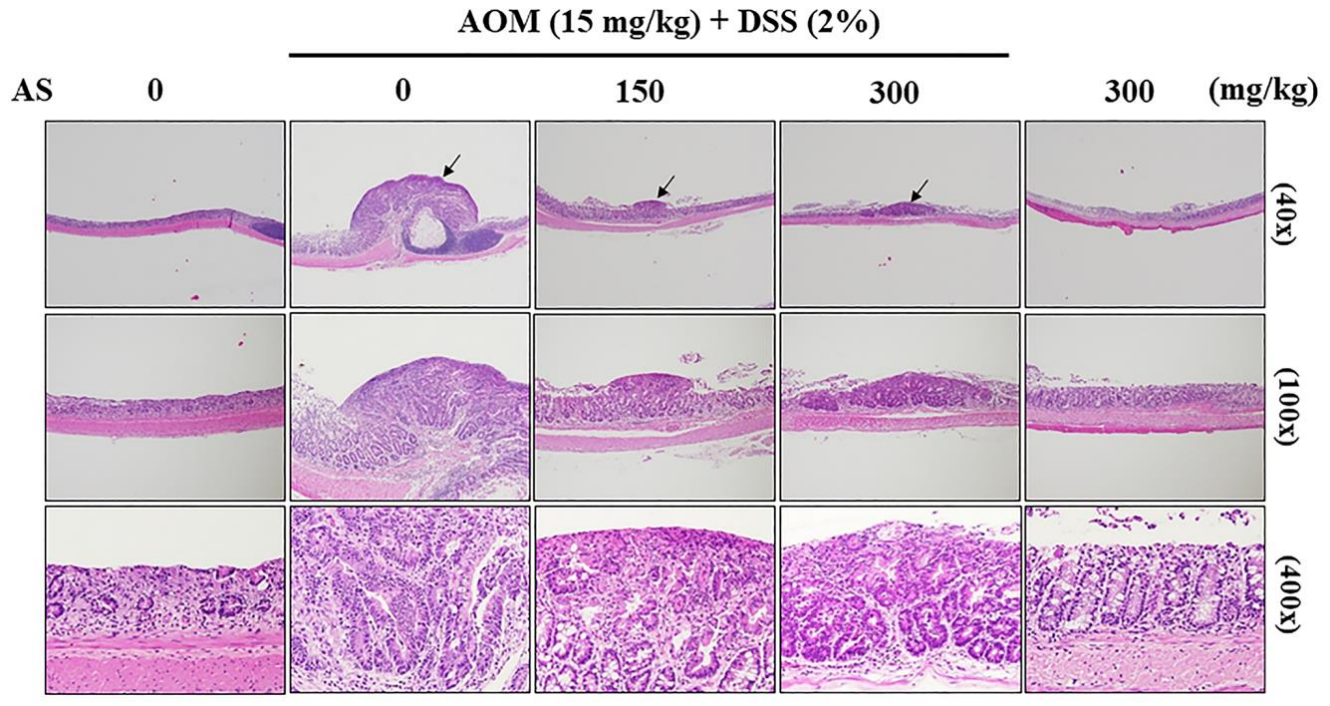
**Histopathological examination of the colon from the AOM/DSS- and/or AS-treated ICR mice.** Representative portion of colon tissues was stained by hematoxylin and eosin, and observed under 40×, 100×, and 400× magnification.

To examine the pathology of colons which were induced by AOM/DSS, colon tissues were stained with hematoxylin and eosin which showed abundant mitosis and CAC was observed in the AOM/DSS group, while AS (150 and 300 mg/kg considerably inhibited the progression of CAC that was induced by AOM/DSS (Figure 14). These findings suggested that AS had powerful effects on suppression of CAC in AOM/DSS-treated mice.

### AS suppresses inflammatory regulatory proteins expression

**Figure 15:**
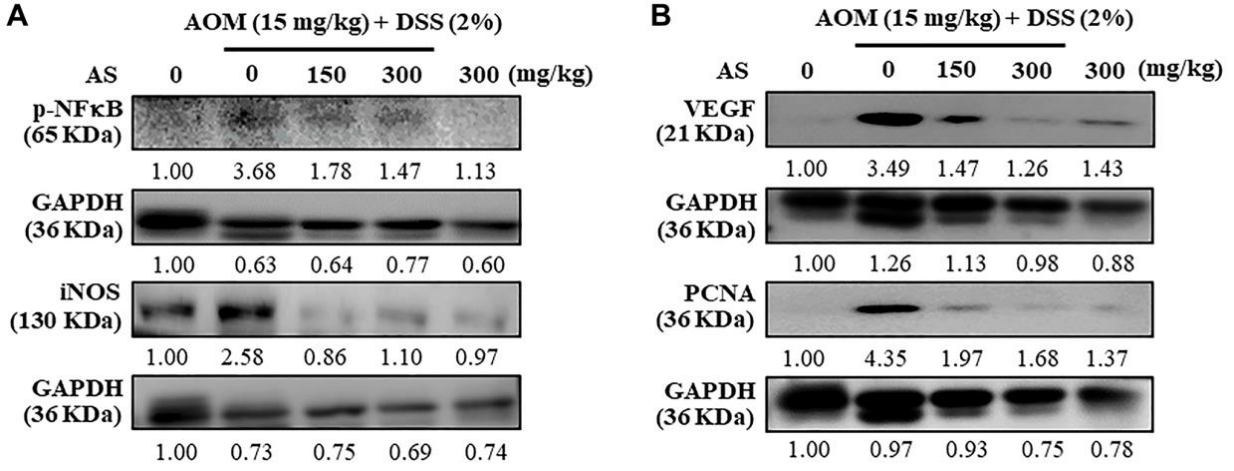
**Mice were injected intraperitoneally with a single dose (15 mg/kg) of AOM (the first week) followed by 3 times of 2% DSS given in the drinking water every two days (the second week).** AS (150 and 300 mg/kg) was given oral administration every two days. Mice were sacrificed on 15 weeks after CAC induction and the expression of (**A**) p-NFκB, iNOS (**B**) VEGF and PCNA were analyzed using Western blotting.

Mice were injected intraperitoneally with a single dose (15 mg/kg) of AOM in the first week followed by 3 times of 2% DSS given in the drinking water every two days in the second week. AS (150 and 300 mg/kg) was given oral administration every two days and sacrificed in 15 weeks and the levels of inflammatory and angiogenesis regulators like p-NFκB, iNOS, VEGF and PCNA were measured using Western blotting. The results revealed that AS (150 and 300 mg/kg) treated mice along with AOM and DSS showed decreased expression of these proteins in comparison to mice which were only given AOM/DSS (Figure 15A and 15B) indicating that AS has chemopreventive potential in colon tumorigenesis due to its effects in inflammation and angiogenesis. However, this decreased expression was more significant with the treatment of AS (300 μg/ml).

## DISCUSSION

Number of evidence suggest that natural products or compounds derived from food and plants have potential for better chemopreventive and chemotherapeutic advantages in human cancer [41, 42]. Recently, chemotherapy treatment of cancer has been interrogated due to increasing incidence of tumor relapse and drug resistance [43]. Emerging studies have highlighted the utilization of natural herbal medicines and their bioactive components as an alternative way for the treatment of cancer [44]. Whole plant extracts are found to be more beneficial than the isolated products since there is advantageous interactions between the constituents within them [45]. AS had shown strong antioxidant properties and provide protection against atherogenesis and atherosclerosis [23, 27]. AS induces cell cycle arrest as well as induces apoptosis and autophagy thus exhibiting an anti-proliferative effect in triple negative breast cancer cells [21, 46]. AS shows anticancer activity also in ovarian cancer and human promyelocytic leukemia [24, 26]. This study uncovered the molecular mechanisms in detail that are involved in AS-mediated apoptosis in colon cancer cells. Flow cytometry analysis showed that 0–200 μg/mL treatment of AS for SW620 cells for 24 h increased early and late apoptosis in a dose-dependent manner. Furthermore, Z-VAD-FMK had reversed the viability of SW620 cells suggesting that AS played important role in death of human colon cancer cells by inducing apoptosis. Our findings showed that ERK signaling cascades have mediated AS-induced cytoprotective autophagy and induced apoptosis. Inhibition of the AKT/mTOR and NFκB signaling cascades was consistent with AS induced cytoprotective autophagy. Apoptosis is distinguished by sequential events like morphological changes of cell, condensation of chromatin, cleavage of DNA, and activation of caspase cascades [47]. As validated by TUNEL assays and Western blot tests, AS induced cell death caused by apoptosis was associated with internucleosomal cleavage of DNA, caspase-3, and caspase-9 activation [48].

Anti- and pro-apoptotic proteins like Bcl-2, Bcl-xL, Bak and Bax ruled cell death by apoptosis [34]. From our former observations, we noted that AS apoptotic induction is correlated with Bcl-2 down-regulation and Bax up-regulation in the cell lines of human breast cancer [21]. Likewise, AS treatment reduced Bcl-2 and enhanced Bax in human ovarian cancer cell line SKOV-3 [24]. All these findings suggested that treatment of AS to cancer cells disrupts the Bcl-2/Bax ratio thus leading to apoptosis.

In the presence of dATP, cytosolic cytochrome c binds with Apaf1 and then activates procaspase-9 thus activating caspase-9. The downstream effector caspases like caspase-3 are gradually activated and then apoptosis is triggered [49]. In most eukaryotic cells PARP is found down-stream of caspase-3 and stimulated during apoptosis [50]. This study revealed that caspase-3 activation and PARP degradation occurred due to AS treatment providing a powerful evidence that apoptosis was enhanced by AS in SW620 cells. Anti-apoptotic proteins like Bcl-2 and Bcl-xL, are included in the Bcl-2 gene family and are believed to be involved in resistance to traditional way of treatment of cancer. However, pro-apoptotic proteins such as Bax, Bak and Bad from the same gene family, can cause apoptotic cell death [51]. In SW620 cells, treatment of AS remarkably diminished the Bcl-2 and increased Bax. Due to this effect, AS treatment exacerbate Bcl-2/Bax ratio inducing apoptosis. This finding thus proved that mitochondrial mediated intrinsic pathway of apoptosis was induced by the treatment of AS in colon cancer cells. Recently, induction of apoptosis becomes a prominent strategy for cancer treatment [52].

In mitochondria, due to oxidative phosphorylation ROS are produced. ROS is highly reactive molecule with unpaired electron in its outermost shell and induced apoptosis because of the formation of superoxide anion (O_2_^•−^) as well as hydrogen peroxide (H_2_O_2_) [53]. Increased levels of ROS are generated due to increased metabolic activity in cancer cells [54]. Oxidative stress means an increasing levels of intracellular reactive oxygen species (ROS) which destruct lipids, proteins and DNA [55]. Deregulations in levels of ROS and autophagy play a crucial role in the development and beginning of cancer and regarded as probable targets for treatment of cancer [56]. Autophagy has double role in tumor suppression as well as promotion in many form of cancers hence acting as possible therapeutics for cancer [57].

One of the important characteristics of autophagy is the formation of AVOs and LC3-II aggregation which is closely related with the number of autophagosomes [58]. Enormous formation of AVOs and LC3-II accumulation provided the clear evidence that due to treatment of AS in SW620 cells activation of autophagy occurred. p62/SQSTM1 is a multidomain protein that is interrelated with autophagy and involved in different cellular processes [59]. The expression of p62/SQSTM1 was increased with increasing concentration of AS indicating that AS-induced autophagy in SW620 cells. We noted that Z-VAD-FMK induced apoptosis inhibition didn’t show any effects on CoQ_0_-induced autophagy (LC3-II accumulation). When the cells were treated with autophagy inhibitors 3-MA (1.5 mM) and CQ (10 μM) along with AS it showed that 3-MA reduced AS-induced AVOs formation however CQ increased the appearance of AVOs. Thus, these results indicated that there were events of early and late autophagy of SW620 cells. Additionally, the transformation of LC3-I to LC3-II was enhanced by the treatment of AS increasing time with maximum conversion at 12 h time which suggested that AS first induce autophagy and then apoptosis.

AS remarkably decreased ATG4B expression in SW620 cells compared to control cells when treated in dose-dependent fashion thus suggesting that there was AS-induced ROS generation in regulation of ATG4B levels in SW620 cells. A tremendous amount of reports suggest that ATG4B expression is elevated in various kinds of cancer, indicating that ATG4B is a potential anticancer target [60]. When SW620 cells was treated with NAC, (1 mM) an ROS inhibitor, to know whether AS-induced apoptosis/autophagy was dependent with ROS generation, cells pretreated with NAC significantly rescued the AS-induced cell death. Similarly, NAC showed inhibitory effects on AS-induced caspase-3 and LC3-II accumulation. Cells treated with AS induced AVOs formation, whereas NAC pre-treated cells showed significantly reduced AVOs formation in SW620 cells. These findings confirm that AS induced ROS-mediated apoptosis and autophagy in human colon cancer SW620 cells.

The ERK signaling pathway plays an important function while regulating different cellular processes like proliferation, differentiation, development, survival, and even apoptosis [61]. In this study, AS activated p-ERK in SW620 cells in time dependent manner. When SW620 cells were pretreated with ERK inhibitor PD98059 and AS, there was significant increment in caspase-3 activation however LC3-I/II accumulation was comparatively decreased. Besides, SW620 cells treated with both AS and PD98059 exhibited remarkable reduction of cell numbers when compared to AS alone treated cells thus suggesting that ERK activation is important in AS-mediated apoptosis and cytoprotective autophagy.

Substantial researches revealed that AKT and mTOR played a central role in regulating many basic activities of cells including autophagy and AKT/mTOR dysregulation caused diseases like cancer and diabetes [62]. In this study, it was noted that AS treatment reduced the phosphorylation of AKT and mTOR. NFκB in SW620 cells suggested that AS inhibited AKT/mTOR signaling cascades, NFκB and β-catenin expression levels thus activating cytoprotective autophagy in human colon cancer cells.

Present study showed that treatment of SW620 cells by AS notably induced DCF fluorescence thus indicating the generation of intracellular ROS. By causing mitochondrial membrane damage, increased ROS levels can control the activity of specific proteins involved in the cell death pathway [63]. These results are in line with the findings of the current study, which determined that AS treatment induced growth suppression and generation of ROS in SW620 cells, showing that the reason behind apoptosis was probably because of development of ROS.

Recent researches have revealed that a some of the natural drugs have potent *in vitro* or *in vivo* anti-tumor properties. In addition, beginning with herbal resources, a different variety of bioactive compounds may be produced to be used as chemotherapeutic agents. Bioactive ingredients isolated from *Antrodia salmonea* mainly polysaccharides, triterpenoids, maleic/succinic acid, erogostanes, quinones, and benzenoids need wider assistance [64]. Fermented broth from submerged culture of *Antrodia salmonea* induced cell cycle arrest and suppressed tumor growth in triple negative breast cancer [46]. *Antrodia salmonea* acted as potential chemopreventive agent by activating ROS-mediated cell death in ovarian cancer cells [65]. In the current research, we showed that AS’s fermented culture broth displayed substantial anti-cancer activity against SW620 cells that was mediated by apoptosis induction. Eventually, it is a rational to propose that AS metabolizes the culture medium so that active compounds are released in submerged culture during fermentation. However, compounds derived from AS submerged culture broth should be properly identified and purified for using as an anti-cancer agent.

Recently, AOM/DSS animal induction model has become a representative prevention experimental induction model and is in application because of its similarity with the pathogenic mechanism and clinical symptoms of human colorectal cancer [66]. Chronic inflammation in colon leads to the risk of developing colitis-associated cancer (CAC) [67]. Being a pro-carcinogen, through the formation of O6-methylguanine AOM exerts carcinogenesis in colon [68] whereas DSS is a polysaccharide alike to heparin that dissolves in drinking water and causes colonic epithelial damage causing colitis [69]. In this study, the effect of AS in the inhibition of AOM/DSS-induced colitis and colon tumorigenesis was demonstrated and hence fundamental mechanisms were investigated. Results showed that AS suppressed experimental CAC thus resulting in gross depletion of colitis and colon tumorigenesis as well as increasing colon weight/decreasing colon length, and increasing weight to length ratio in AOM/DSS-induced mice. Likewise, tumor numbers and size were reduced. HE staining revealed that AS suppressed CAC and exerted anti-tumor activity in AOM/DSS-treated mice. Natural products contain various phytochemicals that help to treat cancer growth by blocking various signaling pathways [70]. The level of proliferating cell nuclear antigen (PCNA) expression in glioma was downregulated by Astragaloside IV, a major constituent of *Astragalus membranaceus* [71]. Colitis-associated colon carcinogenesis in mice was inhibited by *Aster glehni*, a common dietary herb, and this chemopreventive effect was strongly mediated by suppression of the NF-kB activation via phosphorylation [72]. Previous research has shown that Silibinin, a flavolignan isolated from *Silybum marianum*, influences the levels of inflammatory and angiogenic mediators such as iNOS and VEGF in colon tumorigenesis when tested in the AOM mouse model [73]. Corroborating with the previous studies, our study also showed the level of expression of inflammatory mediator proteins such as iNOS, VEGF and PCNA were suppressed in AS treated mice. This study therefore offers a basis for using AS in treating and preventing CAC induced colon tumorigenesis.

In conclusion, all the results revealed that *Antrodia salmonea* potentially acted as inducer of cytoprotective autophagy and apoptosis by the regulation of ROS in colon cancer cells. *In vivo* data revealed that AS reduced colitis-associated tumorigenesis in AOM/DSS treated mice. In addition, this is the leading research to provide insight about *Antrodia salmonea* as an important source of fungi for treatment of human colon cancer. These findings could provide a model of the effects of *Antrodia salmonea* for possible studies of large animal as well as human and thus promote the production of its nutraceutical products.

## MATERIALS AND METHODS

### Chemicals and reagents

Dulbecco’s Modified Eagle’s Medium-high glucose (DMEM/H), fetal bovine serum (FBS), glutamine, and penicillin/streptomycin were bought from GIBCO BRL (Grand Island, NY, USA). Essential antibodies against BCL2 associated X (Bax), B-cell lymphoma 2 (Bcl-2), and β-actin were obtained from Santa Cruz Biotechnology, Inc. (Heidelberg, Germany). Essential antibodies against caspase-3, poly ADP ribose polymerase (PARP), microtubule-associated protein light chain 3B (LC3B), sequestosome 1 (p62), autophagy related 4B cysteine peptidase (ATG4B), Beclin-1, serine/threonine kinase (AKT), p-AKT (Ser473), mammalian target of rapamycin (mTOR), p-mTOR (Ser2448), inducible nitric oxide synthase 1 (iNOS), vascular endothelial growth factor (VEGF), proliferating cell nuclear antigen (PCNA), and β-catenin were obtained from Cell Signaling Technology, Inc. (Danvers, MA, USA). Antibodies against nuclear factor kappa B (NFκB) were procured from GeneTex (San Antonio, TX, USA). Antibodies against extracellular signal regulated kinase (ERK) and p-ERK1/2 (Thr202/Thr204) were acquired from BD Biosciences (NJ, USA). 4′, 6-Diamidino-2-phenylindole dihydrochloride (DAPI) and ERK1/2 signaling inhibitor (PD98059) were bought from Calbiochem (La Jolla, CA, USA). 3-(4,5-dimethylthiazol-2-yl)-2,5-diphenyltetrazolium bromide (MTT), 2′-7′ dihydrofluorescein diacetate (DCFH2-DA), fluorescein isothiocyanate (FITC), propidium iodide (PI), *N*-acetylcysteine (NAC), 3-Methyladenine (3-MA), and chloroquine (CQ) were purchased from Sigma-Aldrich Chemical Co. (St. Louis, MO, USA). Z-Val-Ala-Asp-fluoromethylketone (Z-VAD-FMK) was bought from Calbiochem (San Diego, CA, USA). Other chemicals were reagent grade or high performance liquid chromatography (HPLC) grade and were procured from either Merck and Co., Inc., (Darmstadt, Germany) or by Sigma-Aldrich.

### Preparation of *Antrodia salmonea* submerged culture

The strain of the *Antrodia salmonea* **(**AS) was isolated and recognized by Dr. Timid Yuan Hwang from Endemic Species Research Institute, Nantou, Taiwan. A voucher specimen (No. AS001) was placed in China Medical University, Taichung, Taiwan. The hyphae of AS and fruiting bodies were set apart. The entire fungi was cut and placed inside a flask with 50 mL of sterile water for homogenization and then suspension obtained from mycelia was cultured in medium with 2.0% glucose, 0.1% wheat powder, and 0.1% peptone (pH 5.0). A suspension culture was allowed to grow in a 2 L Erlenmeyer jar with 1 L of medium by shaking at 120 rpm at 25°C for 10 days. For this, culture beaker of 3.5 L was inoculated and shook into a 500 L fermenting tank holding 300 L of culture medium at 25°C for 30 days. The fermentation procedures were similar to the seed fermentation, however an aeration rate of 0.075 vvm was used to obtain a mucilaginous medium that contained the mycelia. Experiments were carried out in 2 to 4 groups of the totally fermented culture of AS. The intense yellow fermentation product was thickened using a vacuum solidify dryer. The dry matter residue of the matured culture was approximately 15 g/L. The lyophilized sample was then crushed, shook, mixed with distilled water, and then centrifuged at 3000 × g for 5 min, and filtered along a 0.22 μm filter. The aqueous extract thus obtained was concentrated using a vacuum freeze dryer to get the powder form [21, 26]. About 0.375 g yield was obtained from 1 g of fermented culture broth of AS. For preparing the stock solution, AS powder was mixed with 10 mM sodium phosphate buffer having pH 7.4 with 0.15 M NaCl (PBS) at 25°C. The mixture was kept at –20°C until further uses.

### Cell culture and treatment

Three human colon cancer cell lines; SW620, HCT116, and HT-29 were purchased from the American Type Culture Collection (Manassas, VA, USA) and were grown in DMEM/H medium which was supplemented along with 10% heat-inactivated FBS, 2 mM L-glutamine, and 1% penicillin-streptomycin in a humidified incubator maintained at 37°C with 5% CO_2_. Cells were collected, and their morphology was inspected through phase-contrast microscopy. Number of cells were counted by means of a hemocytometer (Marienfeld, Germany). Cells were treated with differing amounts of AS (0–300 μg/mL) and the culture time was depended on the concentration. The cells were pretreated with pharmacological inhibitors like Z-VAD-FMK (20 μM), 3-MA (1.5 mM), CQ (10 μM), or NAC (1 mM) for 1 h, and incubated with the demonstrated concentrations of AS for 24 h.

### Analysis of cell viability through MTT assay

The influence of AS on the viability of human colon cancer (SW620, HCT116, and HT-29) cells was examined via the MTT method [74]. Briefly, 3 × 10^4^ cells per well were grown in 24-well plates and treated with different amounts of AS (0–300 μg/mL) for 24 h. Earlier to AS treatment, 400 μL of 0.5 mg/mL MTT in PBS was incubated in each well. After culturing at 37°C for 2 h, 400 μL of DMSO were used to dissolve up the MTT formazan crystals, and the absorbance was monitored at 570 nm (A570) by utilizing ELISA microplate reader (μ-Quant, USA). Cell viability rate (%) was determined as (A570 of treated cells/A570 of untreated cells) × 100. The experiments were carried out in triplicates.

### Assessment of ROS generation

ROS that were generated intracellularly was recognized by fluorescence microscopy employing DCFH_2_-DA which is a fluorogenic dye [21] permeable to cells. 7 × 10^5^ cells were cultured in 6-cm dish in DMEM/H medium which was supplemented with 10% FBS. When the cells reached 80% confluency, the culture medium was supplanted. In order to know the ROS formation in a time-dependent manner, the cells were provided with 200 μg/mL of AS for 0–120 min and then the supernatant was thrown out and cultured together with non-fluorescent DCFH_2_-DA (10 μM) in a freshly prepared medium at 37°C for 30 min. The intracellular aggregation of dichlorofluorescein (DCF) due to the oxidation of DCFH_2_ represented the level of intracellular ROS. The DCF-stained cells were photographed using phase contrast fluorescence microscopy at 200× magnification (Olympus, Center Valley, USA). The fluorescence was calculated by using LS 5.0 (Olympus Imaging America Inc., USA). The changes in fold in fluorescence intensity i.e. ROS generation was arbitrarily assigned as 1 in comparison to the untreated control cells.

### Determination of apoptotic cells by Annexin V/PI staining

SW620 cells were stained by Annexin V-FITC and PI to estimate the rate of apoptosis [21, 26]. In short, at first different concentrations of AS (0, 100, 150, or 200 μg/mL) were used to treat the cells for 24 h. The cells were later trypsinized and washed with PBS two times, and finally centrifuged at 1000 rpm for 5 min. 7 × 10^5^ cells in 6-cm dish were suspended in 500 μL of standard buffer and then double-stained with Annexin V-FITC along with PI for 15 min at room temperature. The fluorescence intensity of the FITC (green) and PI (red) of every test was measured quantitatively by FACS Calibur Flow Cytometry (Becton Dickinson, USA) and CellQuest software. The results of flow cytometry were determined using four quadrants; Necrotic cells in Q1 with PI-positive and Annexin V-FITC-negative; late apoptotic cells in Q2 with PI-positive and Annexin V-FITC-positive; normal viable cells in Q3 with both PI and Annexin V-FITC negative, and early apoptotic cells in Q4 with PI-negative and Annexin V- FITC-positive.

### Mitochondrial functional assay

Fluorescent imaging of mitochondria was performed by using a Mito-Tracker Green detection kit (Molecular Probe, Eugene, OR) by properly following the instructions from the manufacturer. Mito-Tracker is a type of green fluorescent dye that localizes the mitochondria despite of mitochondrial membrane potential. 2 × 10^4^ SW620 cells per well were grown on eight-well Tek chambers and exposed to various concentrations of AS (0, 100, 150, and 200 μg/mL) for 24 h. Post AS treatment, fixation of cells was done using 2% paraformaldehyde in PBS solution for 15 min and later incubated along with 1 μM Mito-Tracker for 30 min and then stained with 1 μg/mL DAPI for 5 min, and observed using a fluorescence microscope at 400× magnification.

### Protein isolation and immunoblotting

2 × 10^6^ cells in 10-cm dish were grown with AS (0–200 μg/mL) as specified for the definite time. The cells were collected, pooled, washed one time with PBS after culture, and mixed in 89 μL of lysis buffer which contained 10 mM Tris-HCl with pH 8, 32 mM sucrose, 1% Triton X-100, 5 mM EDTA, 2 mM DTT, and 1 mM PMSF. Thus obtained lysates were kept on ice for 30 min and afterwards centrifuged at 12,000 rpm for 30 min at 4°C. Protein test reagent (Bio-Rad, Hercules, CA, USA) was utilized to determine the level of total protein by taking BSA as standard. The protein extract was dissolved in buffer with 62 mM Tris-HCl, 2% SDS, 10% glycerol, and 5% β-mercaptoethanol. After that, the mixture was heated for 5 min at 97°C. Equal volume (50 μg) of denatured protein were allowed to 8–15% SDS-PAGE and moved over polyvinylidene difluoride (PVDF) membranes, and left overnight. For blocking, the membranes were allowed to incubate with 5% non-fat dry milk in PBS with 1% Tween-20 for 1 h at room temperature and later with primary antibodies for overnight. 2 h prior to image processing that utilized a chemiluminescent substrate (Millipore, USA), the membranes were incubated with secondary antibodies; either a horseradish peroxidase (HRP) conjugated anti-rabbit or anti-mouse antibody. The changes in protein expression levels were evaluated by an ImageQuant™ LAS 4000 (Fujifilm). Densitometric examinations were carried out using commercially available quantitative software (AlphaEase, USA), with the control that represented 1-fold, and in protein levels were shown in the histograms.

### Detection of acidic vesicular organelles

Acridine orange (AO) stain was used to examine the formation of acidic vesicular organelles (AVOs) in SW620 cells [75]. 7 × 10^5^ cells in 6-cm dish were treated with AS, and then followed by washing two times with PBS. Later the cells were stained with AO (1 μg/mL), diluted, washed, and then covered in PBS containing 5% FBS. The formation of AVOs in cells were detected by observing under a red filter of fluorescence microscope.

### Experimental mice

5-week old male Institute of Cancer Research (ICR) mice were brought from the National Laboratory Animal Center (Taipei, Taiwan) and were kept in aseptic conditions of a 12–12 h light-dark cycle by feeding rodent chow (Oriental Yeast Co., Tokyo, Japan) as well as by supplying unlimited access to water. All protocols involving animals and their welfare were approved by China Medical University’s Institutional Animal Care and Treatment Committee. Every effort was made to minimize animals' number and to alleviate the suffering of the animals.

### Colitis-associated carcinogenesis induction through AOM/DSS and AS treatment

For carrying out induction of colitis-associated cancer (CAC) and its treatment through AS, BALB/c mice were grouped into five different classes (*n* = 9~13). Group I mice were treated as control. Group II mice were injected intraperitoneally with a single dose (15 mg/kg) of Azoxymethane (AOM) for 1 week and then followed by 2% Dextran sulfate sodium (DSS) along with drinking water for three times every two days for second week to induce CAC. Group III and IV mice were orally given 150–300 mg/kg of AS along with AOM and DSS with drinking water for every two days, respectively. Group V mice were provided with oral administration of AS (300 mg/kg) for every two days. The body weight of each mice was measured every week to inspect drug toxicity All mice were sacrificed on 15 weeks after CAC induction and the different organs of body like liver, kidney, and spleen were weighed to observe any possible side effects of the treatment.

### Histopathological analysis

Prior to the fixation in 10% neutral buffered formalin the whole colon was removed and the length and weight was measured. Then sectioning and hematoxylin-eosin staining was performed followed by light microscopic analysis (Olympus, Center Valley, PA, USA). At first, using 10% neutral buffered formalin excised xenografted tumors were fixed for 2 days and then rinsed with saline and dehydrated with a series of 50%, 70% and 95% of ethanol. Thus acquired dehydrated samples were implanted in paraffin and sliced into 5 μm thick sections. Later sections were deparaffinized in xylene solution and rehydrated in series of alcohol as described earlier. For blocking of endogenous peroxidase, the sections were immersed in 3% (v/v) hydrogen peroxide in methanol for 15 min and then washed two times with PBS. The tumor tissues sections were analysed by a veterinary pathologist to examine cells in mitotic state post AS treatment comparing to control mice sections. The biopsied tumor tissues were inserted in paraffin block, sectioned into 3-mm-thickness, set down in plastic cassettes, and kept immersing in formalin containing neutral buffer for 24 h. Thus obtained fixed tissues were later deparaffinized and again hydrated. Then by utilizing standard techniques, the morphological and intracellular changes were examined in colon parts stained with hematoxylin and eosin and the extent to which treatment with AS could inhibit the progression of colon cancer was assessed.

### Statistical analysis

The results are expressed as the mean ± standard deviation (mean ± SD) or mean ± standard error of mean (mean ± SEM) (*n* = 9~13). The obtained data were inspected by analysis of variance (ANOVA), and accompanied by Dunnett’s test for pair wise comparison. Statistical significance was defined as ^*^p < 0.05, ^**^p < 0.01, and ^***^p < 0.001 compared to the untreated control. ^#^*p* < 0.05, ^##^*p* < 0.01, and ^###^*p* < 0.001 compared with AS-treated cells or AOM/DSS-treated cells.
